# Identification of a Novel Fungal α‑l‑Fucosidase with Transfucosylation Potential

**DOI:** 10.1021/acsomega.6c00254

**Published:** 2026-08-04

**Authors:** Pavlína Nekvasilová, Michaela Glozlová, Andrea Vopálenská, Lucie Petrásková, Natalia Kulik, Pavla Bojarová

**Affiliations:** † 48311 Institute of Microbiology of the Czech Academy of Sciences, Vídeňská 1083, Prague 4 CZ-14200, Czech Republic; ‡ Department of Genetics and Microbiology, Faculty of Science, Charles University, Viničná 5, Prague 2 CZ-12843, Czech Republic; § Department of Organic Chemistry, Faculty of Science, Charles University, Hlavova 2030/8, Prague 2 CZ-12800, Czech Republic

## Abstract

Fucosylated carbohydrates are a vital part of human nutrition. They act as prebiotics, nourishing beneficial gut bacteria and helping shape the gut microbiome. Enzymatic synthesis is a convenient method to access these molecules. Fungal α-l-fucosidases are glycoside hydrolases that naturally cleave terminal α-linked fucose residues from glycans; those with synthetic capabilities are conveniently applicable in glycoengineering. This study investigates the fucosylation potential and regioselectivity of rare GH29 α-l-fucosidases from filamentous fungi. The screening of production of putative α-l-fucosidases by fungal strains using various inducers was combined with in silico analysis. Recombinant α-l-fucosidases were produced on a large scale and characterized with respect to their substrate specificities and pH optima. A novel transfucosylating α-l-fucosidase from *Aspergillus phoenicis* was thus identified and characterized. It was capable of regioselective formation of an α-(1 → 2)-linked fucosylated product. These findings highlight the potential of selected fungal α-l-fucosidases as promising tools for glycoengineering of fucosylated carbohydrates.

## Graphical abstract

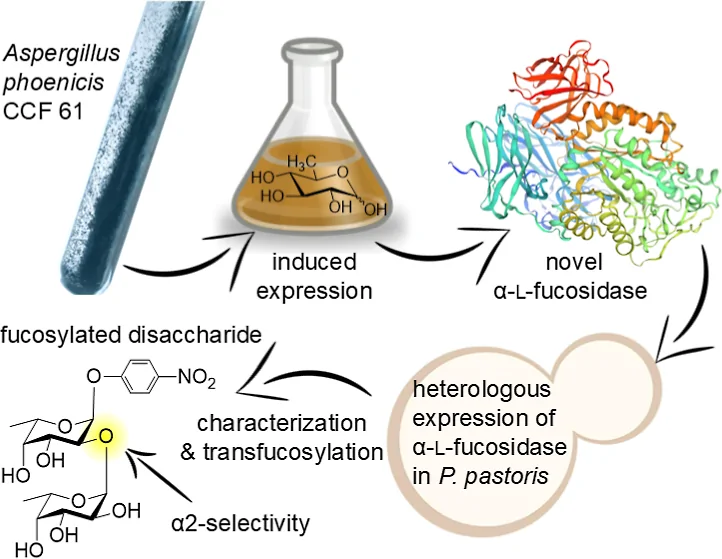

## Introduction

Fucosylated carbohydrates represent an important group of bioactive carbohydrates with a key role in human nutrition and food chemistry. They are naturally present in human breast milk, where they constitute up to 60% of all oligosaccharides, and exhibit a number of beneficial effects: they act as prebiotics, modulate the intestinal microbiome, support the development of the immune system, and exhibit antiadhesive and antimicrobial properties.^1,2^ Fucosylated carbohydrates are intensively studied for their health benefits in infants, including support for cognitive development and protection against pathogens.^3^ These properties make these molecules attractive targets for the development of functional foods, nutraceuticals, and infant formulas.

Due to their complex structure and low availability from natural sources, the synthesis of fucosylated carbohydrates using enzymes, particularly fucosyltransferases and α-l-αfucosidases, is considered a promising route for their production.^4^ Enzymatic methods allow for precise regio- and stereoselective synthesis under mild conditions, advantageous for food applications.

Bioactive fucosylated oligosaccharides are naturally synthesized by fucosyltransferases—essential enzymes for the biosynthesis of fucosylated human milk oligosaccharides (HMO) in vivo. These enzymes catalyze the transfer of l-fucose from GDP-fucose to specific positions on lactose-based oligosaccharides,^5,6^ and can be applied in biocatalytic cascades to produce HMOs in vitro.^7^ They are typical of high regio- and stereoselectivity, mild reaction conditions, and a strong potential of reaction scalability.^8^ However, their limitations include the availability of GDP-fucose, and acceptor specificity.^9^ Therefore, synthetically potent α-l-fucosidases emerge as a prospective alternative for the synthesis of fucosylated target structures.^10,11^

α-l-Fucosidases belong to *exo*-glycosidases (EC 3.2.1.-), catalyzing the hydrolysis of an α-linked fucosyl residue from nonreducing end of glycans.^9,12^ According to their primary sequence similarities and catalytic mechanism, they can be classified in five glycoside hydrolase families (GH families) in the Carbohydrate Active Enzymes database (CAZy; http://www.cazy.org/).^13^ Most α-l-fucosidases are included in two families, GH29 and GH95, but there are also the relatively newly established families GH139, GH141 and GH151.^14,15^

GH29 α-l-fucosidases are found across different domains of living organisms, including bacteria, fungi, plants, and animals. According to the CAZy database (October 2025), 16,645 α-l-fucosidases are annotated in GH29 family, of which, however, solely five fungal α-l-fucosidases are characterized^16–19^ and only two have a resolved crystal structure.^20,21^ GH29 α-l-fucosidases are retaining glycosidases utilizing the classical Koshland double-displacement reaction mechanism.^22–24^ When a nucleophile different from water enters the active site and attacks a glycosyl-enzyme intermediate, GH29 α-l-fucosidases can also catalyze transfucosylation with a suitable fucosyl donor.^25^ Thus, there is a competition between transfucosylation and hydrolysis under aqueous solutions. Generally, the reaction conditions can be optimized in favor of transfucosylation by, for example, a high acceptor/donor ratio^26^ or by adding an organic solvent,^27^ although in some cases the required conditions are extreme, such as 1000-fold molar excess of donor over acceptor,^26^ and the yields are rather poor.

GH29 α-l-fucosidases exhibit a broad substrate specificity, acting on α-(1 → 2), α-(1 → 3), α-(1 → 4), and α-(1 → 6)-fucosyl linkages. Based on their sequence identity and substrate specificity, these enzymes are classified into two subfamilies: GH29A and GH29B.^28^ In general, GH29A α-l-fucosidases show higher activity with synthetic chromogenic substrates, such as 4-nitrophenyl α-l-fucopyranoside (*p*NP-α-l-Fuc) and 2-chloro-4-nitrophenyl α-l-fucopyranoside (CNP-α-l-Fuc). In contrast, GH29B α-l-fucosidases are often inactive with these synthetic substrates,^29^ which renders GH29A α-l-fucosidases more suitable for synthetic purposes.

This study explores the properties, production, and synthetic potential of novel, yet unexplored α-l-fucosidases from filamentous fungi with the aim to identify prospective representatives for the synthesis of fucosylated carbohydrates. Novel fungal α-l-fucosidases were identified through bioinformatics, produced heterologously in *Pichia pastoris*, and characterized for their biochemical properties, including their transfucosylation activity. The study highlights the potential of these enzymes in the synthesis of fucosylated carbohydrates for therapeutic applications.

## Materials and Methods

### In Silico Analysis

The in silico search after new fungal α-l-fucosidases was performed with BLAST (Basic Local Alignment Search Tool, https://blast.ncbi.nlm.nih.gov/Blast.cgi; National Center for Biotechnology Information).^30^ Amino acid sequences of reference α-l-fucosidases, namely α-l-fucosidase from the filamentous fungus *Fusarium graminearum* (*Fg*FCO1, AFR68935.1)^20^ and from the bacterium *Thermotoga maritima* (*Tm*αFuc, AAD35394.1)^23^ were aligned with amino acid sequences of putative enzymes in the BLAST tool. The reference enzymes were chosen for their known α2-specificity and established transfucosylation ability. To expand the choice of these rare enzymes in fungi for our study, candidate sequences were selected based on minimum requirements—moderate sequence similarity (typically ≥25% identity), query coverage ≥70%, and conservation of key catalytic residues of GH29 α-l-fucosidases, with preference given to taxonomically diverse fungal species.

The phylogenetic relationship of putative α-l-fucosidases from *Apiotrichum porosum*, *Absidia repens*, and *Clonostachys rhizophaga*, selected based on their sequence homology to reference α-l-fucosidases *Tm*αFuc and *Fg*FCO1, was inferred using Molecular Evolutionary Genetics Analysis software (MEGA11).^31^ Amino acid sequences of selected enzymes were translated into nucleotide sequences using program SMS: Reverse Translate (Sequence Manipulation Suite, https://www.bioinformatics.org/sms2/rev_trans.html).^32^ The respective genes *apfuc*, *arfuc* and *crfuc* were designed for the insertion into the yeast expression vector pPICZαA using codon preference for *P. pastoris* according to the Codon Usage Database (https://www.kazusa.or.jp/codon/).^33^ The presence of introns was identified by Augustus (https://bioinf.uni-greifswald.de/augustus/).^34^ Amino acid sequences were analyzed for the presence of conserved domains using InterPro (https://www.ebi.ac.uk/interpro/)^35^ and Conserved Domains program by NCBI (https://www.ncbi.nlm.nih.gov/Structure/cdd/wrpsb.cgi).^36^ The presence of transmembrane segments and signal peptides was detected using the program Predict Protein (https://predictprotein.org/)^37^ The designed genes *apfuc*, *arfuc*, and *crfuc* were cloned into the pPICZαA vector using *Eco*RI/*Kpn*I (*apfuc*) or *Eco*RI/*Xho*I (*arfuc* or *crfuc*) restriction sites, downstream of the α-factor that provides extracellular targeting of the enzyme and resistance to zeocin. The plasmids were prepared commercially (Generay, CN).

### Production of α-l-Fucosidases by Filamentous Fungi

The fungal strains (Culture Collection of Fungi, CCF) of *Aspergillus terreus* CCF55, *Fusarium oxysporum* CCF337, *Mortierella alpina* CCF1525, *Fusarium culmorum* CCF2680, *Mucor circinelloides* CCF195, *Aspergillus flavipes* CCF544, *Aspergillus phoenicis* CCF61, *Penicillium funiculosum* CCF1994, *Penicillium purpurogenum* var. *rubrisclerotium* CCF2984, and *Talaromyces flavus* CCF2628 were first cultivated on inclined sporulation agar (malt extract 45 g L^–1^; NaCl 15 g L^–1^; NH_4_NO_3_ 0.44 g L^–1^; MgSO_4_ 0.05 g L^–1^; CuSO_4_ 1.5 mg L^–1^; agar 30 g L^–1^; pH 5.5). Freshly inoculated agar from parent cultures was then cultivated at 28 °C for 7 days. The mycelium was stored at 4 °C until further use.

The mycelium was then harvested by washing with Tween 80 solution (4 mL; 0.1% *v*/*v*), and the resulting spore suspension (0.5 mL) was inoculated into peptone medium (100 mL; yeast extract 0.5 g L^–1^; bacteriological peptone 5.0 g L^–1^; KH_2_PO_4_ 3.0 g L^–1^; NH_4_H_2_PO_4_ 5.0 g L^–1^; casein hydrolysate 7.5 g L^–1^; pH 6.0) with respective inducers in 500 mL Erlenmeyer flasks as described previously.^38^ After sterilization of the medium, sterile MgSO_4_ solution at a final concentration of 10% *w*/*v* and sterile inducer solutions were added in the final concentration of 0.1% *w*/*v* d-arabinose, 0.5% *w*/*v* l-fucose, 4% *w*/*v* d-glucose, or 0.1% *w*/*v* 6-deoxy-d-glucose. Additionally, fucoidan from *Laminaria japonica* (final concentration of 1% *w*/*v*) was selected as a potential inducer used for induction in the work by Shvetsova et al., 2015.^39^ The cultures were cultivated for 14 days, and the undiluted medium was tested for extracellular production of enzyme in a standard activity assay, which showed α-l-fucosidase activity after 7 days. The genes *tpfuc, tffuc* and *aphfuc*, encoding for putative α-l-fucosidases of *P. purpurogenum* (denoted as *Talaromyces* in BLAST, different from CCF), *P. funiculosum* (denoted as *Talaromyces* in BLAST, different from CCF), and *A. phoenicis* that showed the highest activity in the screening of fungal strains, were identified by a combined experimental and bioinformatic approach and were designed for the insertion into the yeast expression vector pPICZαA as described in detail in the Supporting Information, Section S4.1. To identify the respective genes, available genomic and transcriptomic data of the respective strains of *P. purpurogenum*, *P. funiculosum* and *A. phoenicis* were aligned with known α-l-fucosidase from *Aspergillus niger* (XP_001396349.2),^19^ which was the phylogenetically closest known fungal α-l-fucosidase to these strains, using BLAST database. Using this known sequence, we also annotated the genes of *P. purpurogenum* and *P. funiculosum*, which were not annotated in the BLAST database yet. The gene sequences were identified based on sequence similarity, the presence of conserved catalytic residues characteristic for GH29 family, and predicted signal peptides. The sequences were further analyzed by domain analysis using InterPro and NCBI Conserved Domain Database, and signal peptide prediction (see also section “In Silico Analysis”). The resulting encoding sequences were then codon-optimized for the expression in *P. pastoris*, synthesized, and cloned into the pPICZαA vector. The designed genes *tpfuc*, *tffuc*, and *aphfuc* were cloned into the pPICZαA vector using *Not*I/*Xba*I (*tpfuc*), *Eco*RI/*Xba*I (*tffuc*), or *Eco*RI/*Kpn*I (*aphfuc*) restriction sites downstream of the α-factor that provides extracellular targeting of the enzyme and resistance to zeocin.

### Standard Hydrolytic Assay of α-l-Fucosidases

The hydrolytic activity of α-l-fucosidase was measured in an end-point assay with spectrophotometric detection. *p*-Nitrophenyl α-l-fucopyranoside (*p*NP-α-l-Fuc) was used as a substrate for the hydrolytic reaction. The reaction mixture containing *p*NP-α-l-Fuc (2 mM), and McIlvaine buffer (50 mM citric acid/100 mM Na_2_HPO_4_ pH 5.0) was incubated at 35 °C at 850 rpm, and the reaction was started by adding an appropriately diluted enzyme. The reaction was then stopped after 10 min by adding 1 mL of 0.1 M sodium carbonate. *p*-Nitrophenol was cleaved off the substrate during the reaction and its concentration was determined spectrophotometrically at 420 nm. All measurements were performed in triplicate against a reference sample consisting of substrate and buffer.

## Results

### Putative Fungal α-l-Fucosidases Identified by In Silico Analysis

For the identification of putative α-l-fucosidases, we first performed a search through gene databases. BLAST was used to identify putative fungal enzymes with amino acid sequences similar to the α-l-fucosidases known from the literature. As the reference enzymes, we used α-l-fucosidases from the filamentous fungus *F. graminearum* (*Fg*FCO1, AFR68935.1)^20^ or from the bacterium *T. maritima* (*Tm*αFuc, AAD35394.1) characterized previously.^23^ *Tm*αFuc was selected for its α-(1 → 2)-regioselectivity when transferring Fuc to Gal moiety,^40^ which is desirable for potential applications in the synthesis of human milk oligosaccharides, and *Fg*FCO1 was chosen as a known fungal representative of α-l-fucosidases already heterologously produced and also exhibiting α-(1 → 2)-regioselectivity.^41^ Transfucosylation capabilities of both known enzymes were described previously using lactose as an acceptor and *p*NP-α-l-Fuc or xyloglucan from citrus peels as a donor in the reaction catalyzed by *Tm*αFuc or *Fg*FCO1, respectively.^26,41^

The sequence similarity analysis identified three putative α-l-fucosidases: enzymes from *A. porosum* (*Ap*Fuc; 24% identity to *Fg*FCO1 and 42% identity to *Tm*αFuc), *A. repens* (*Ar*Fuc; 24% identity to *Fg*FCO1 and 38% identity to *Tm*αFuc), and *C. rhizophaga* (*Cr*Fuc; 71% identity to *Fg*FCO1 and 27% identity to *Tm*αFuc) exhibited the highest similarity to the reference enzymes (Figure 1). To achieve a higher diversity of the putative enzymes, other representatives of the genus *Fusarium* were excluded from the selection of new strains.

**Figure 1:**
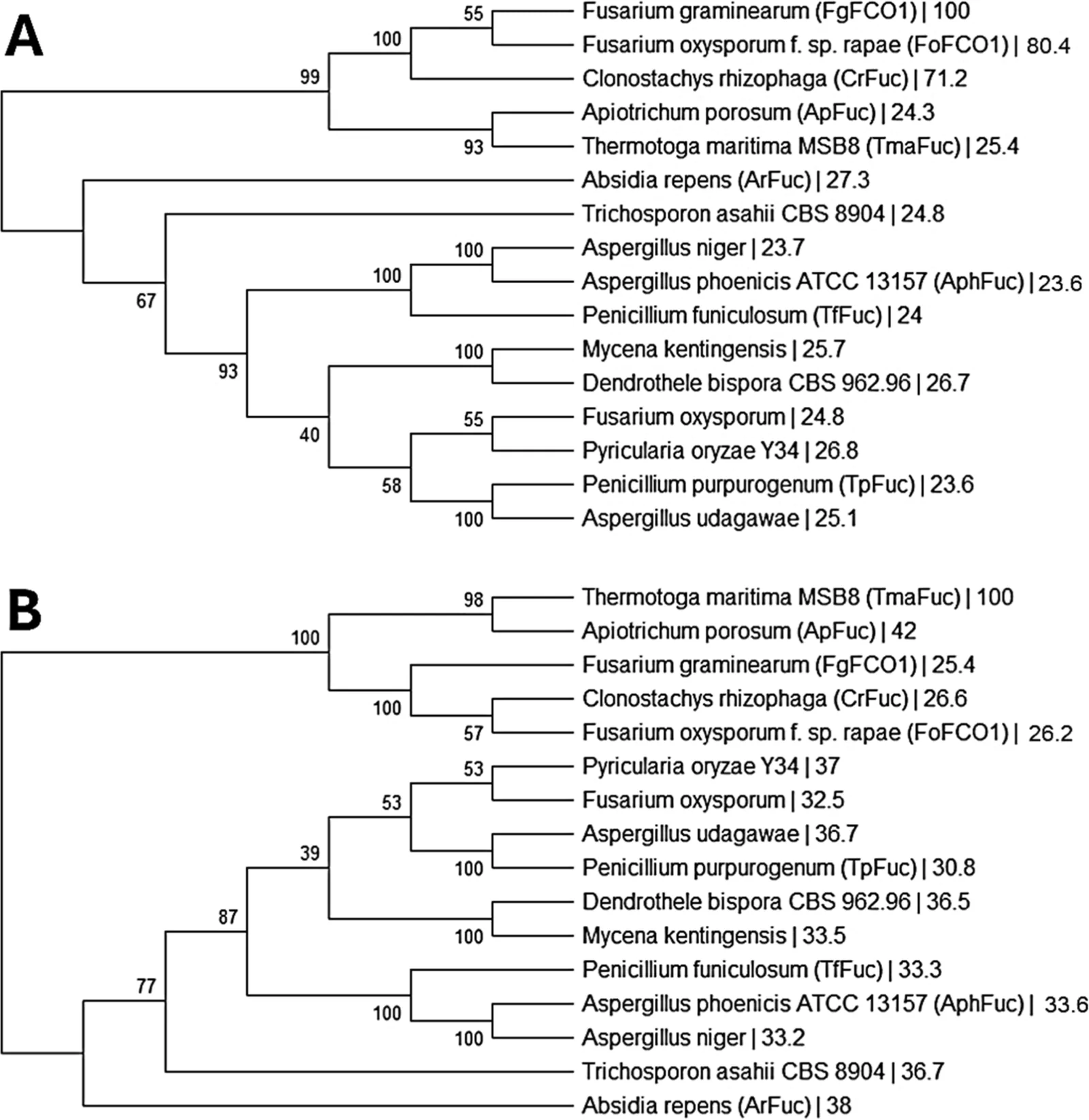
Phylogenetic tree of the compared organisms producing putative α-l-fucosidases using *Fg*FCO1 as a reference α-l-fucosidase (A), or *Tm*αFuc as a reference α-l-fucosidase (B). The relationships between the selected organisms was constructed with Molecular Evolutionary Genetics Analysis software (MEGA11). The enzyme abbreviations used throughout the text were added for clarity.

The synthetic genes of putative enzymes *Ap*Fuc (XP_028471782.1), *Ar*Fuc (ORZ24074.1) and *Cr*Fuc (CAH0042109.1) as annotated in the BLAST database were codon-optimized for the expression in *P. pastoris*. However, despite repeated attempts, none of these three in silico identified putative α-l-fucosidase genes (Table 1, entries 1–3) were successfully transformed into *P. pastoris* and were heterologously expressed. Therefore, we decided to screen for putative α-l-fucosidase activity directly in fungal strains as detailed in the next section.

**Table 1:** Protein Identity of α-l-Fucosidases Designed In Silico or Identified after Induction in Fungal Strains and Known α-l-Fucosidases *Fg*FCO1 and *Tm*αFuc

	sequence similarity [%]	
enzyme (fungal strain)	*Tm*αFuc	*Fg*FCO1
*Ap*Fuc (*Apiotrichum porosum*)^a^	42	24
*Ar*Fuc (*Absidia repens*)^a^	38	24
*Cr*Fuc (*Clonostachys rhizophaga*)^a^	27	71
*Tp*Fuc (*Penicillium purpurogenum*)^b^	31	24
*Tf*Fuc (*Penicillium funiculosum*)^b^	33	24
*Aph*Fuc (*Aspergillus phoenicis*)^b^	34	24

a α-l-Fucosidases identified by in silico analysis.

b α-l-Fucosidases identified by induction in selected fungi (next section).

### Putative Fungal α-l-Fucosidases Identified by Induction in Selected Fungi

From the Culture Collection of Fungi at the Department of Botany, Charles University, Prague, we selected ten representatives, in which α-l-fucosidase activity was expected based on cultivation data from our previous research:^38^ *A. terreus*, *F. oxysporum*, *M. alpina*, *F. culmorum*, *M. circinelloides*, *A. flavipes*, *A. phoenicis*, *P. funiculosum*, *P. purpurogenum* var. *rubrisclerotium*, and *T. flavus*.

We cultured these fungi in peptone medium for 14–21 days under the induction by d-arabinose (0.1% *w*/*v*), l-fucose (0.5% *w*/*v*), d-glucose (4% *w*/*v*), 6-deoxy-d-glucose (0.1% *w*/*v*),^38^ or fucoidan from *L. japonica* (1% *w*/*v*).^39^ Whereas l-fucose and d-arabinose are readily obtainable commercial carbohydrates, 6-deoxy-d-glucose (quinovose; **4**), a prospective inducer in previous experiments^38^ is a relatively expensive commodity. Therefore, for the sake of our screening, we developed a novel synthetic pathway from commercial methyl α-d-glucopyranoside affording this compound in a gram amount in a 23% overall yield (see Supporting Information, Section S3, and Figures S7–S10).

After 6 days of cultivation, the undiluted medium was tested for extracellular α-l-fucosidase activity in a standard activity assay. Activity tests were performed daily until day 14; however, for three representatives induced by 6-deoxy-d-glucose, the activity was measurable only after 13 days of cultivation; therefore, in these cases the cultivation time was extended to 21 days. After the cultivation of selected fungi in peptone medium with inducers, four strains exhibiting α-l-fucosidase activity were identified: *F. oxysporum* after induction with l-fucose and d-arabinose; *P. purpurogenum*, *P. funiculosum* and *A. phoenicis* after induction with 6-deoxy-d-glucose. The results for *P. purpurogenum* and *P. funiculosum* confirmed the findings by Huňková et al.^38^ whereas the results for *A. phoenicis* and *A. flavipes* were different. In the previously mentioned study, these fungi produced enzymes with α-l-fucosidase activity upon induction with 4% *w*/*v* glucose; however, this result was not confirmed by us. Here, α-l-fucosidase activity was detected in the medium of *A. phoenicis* induced by 6-deoxy-d-glucose, and it was not confirmed in *A. flavipes* upon any induction. For the other fungi tested, namely *A. terreus*, *M. alpina*, *F. culmorum*, *M. circinelloides*, and *T. flavus*, no extracellular α-l-fucosidase activity with *p*NP-α-l-Fuc was detected during the screening.

The growth curves of fungi with positive α-l-fucosidase activity are shown in Supporting Information, Figure S1. The graphs illustrate the extracellular α-l-fucosidase activity of various fungal strains in time under various induction conditions. *F. oxysporum* cultivated under the induction by l-fucose (Supporting Information, Figure S1A) exhibited the highest volume enzyme activity, reaching approximately 1.4 U mL^–1^ on day 10 before gradually declining to ca. 1.1 U mL^–1^ by day 14, indicating a strong and transient induction. In contrast, when induced with d-arabinose (Supporting Information, Figure S1B), *F. oxysporum* showed minimal volume activity, peaking at only ∼0.04 U mL^–1^, suggesting weak induction. The third graph (Supporting Information, Figure S1C) depicts three other fungal strains, *P. purpurogenum*, *A. phoenicis*, and *P. funiculosum*, grown under the induction by quinovose (**4**). All these strains displayed delayed enzyme induction, with peak activities observed around day 17. Among them, *P. funiculosum* showed the highest volume activity (∼0.07 U mL^–1^), followed by *A. phoenicis* (∼0.03 U mL^–1^) and *P. purpurogenum* (∼0.02 U mL^–1^). These results demonstrate the variability of α-l-fucosidase production depending on the fungal species and the inducer type.

To avoid lengthy and inefficient production of these enzymes in the natural fungal producer, we opted for their heterologous expression. The sequences encoding for putative α-l-fucosidases from *P. purpurogenum* (*Tp*Fuc), *P. funiculosum* (*Tf*Fuc), and the annotated sequence from *A. phoenicis* (*AphFuc*; RDK40322.1), which all showed the highest volume activity in the screening of fungal strains, were identified by sequence alignment of the genomic and transcriptomic data of these strains available in the BLAST database with the phylogenetically closest α-l-fucosidase from *A. niger* (XP_001396349.2).^19^ *Tp*Fuc, *Tf*Fuc, and *Aph*Fuc shared 42%, 69%, and 97% identity, respectively, with this α-l-fucosidase. Interestingly, as shown in Table 1 (entries 4–6), the protein sequences did not have a substantial similarity to either *Fg*FCO1 or *Tm*αFuc, a criterion used in our previous in silico screening. The genes were codon-optimized for the expression in *P. pastoris* and the introns were excluded using Augustus program.^34^

Two partially characterized enzymes from *F. graminearum* and *F. oxysporum* (*Fo*FCO1, AFR68934.1)^16,42,43^ were added to the selection for comparison.

The analysis of the conserved domains confirmed that all selected sequences contain the GH29 domain typical for α-l-fucosidases. The Predict Protein application^37^ detected one signal peptide in the *Fg*FCO1 and *Fo*FCO1 genes (the sequence encoding for the signal peptide was retained in the sequence), and no transmembrane stretches.

First, the expression of recombinant α-l-fucosidases *Fg*FCO1, *Fo*FCO1, *Tp*Fuc, *Tf*Fuc, and *Aph*Fuc in *P. pastoris* was screened on a small scale. For each enzyme, 16 clones were inoculated and after 5 days of cultivation in complex medium, α-l-fucosidase activity was measured by standard activity assay and the α-l-fucosidase expression was checked by SDS-PAGE. The genes of enzymes *Fg*FCO1, *Fo*FCO1, and *Aph*Fuc were successfully heterologously expressed in *P. pastoris*. In the case of *Tp*Fuc and *Tf*Fuc, protein expression was not observed whatsoever, despite repeated attempts. Therefore, in further research, we concentrated on the three active recombinant enzymes.

### Heterologous Production and Purification of Recombinant Fungal α-l-Fucosidases

Large-scale productions of partially characterized enzymes *Fg*FCO1, *Fo*FCO1, and the new enzyme *Aph*Fuc were performed in minimal cultivation medium to facilitate subsequent protein purification by ion exchange chromatography. The purification process was optimized for each enzyme, testing cation and anion exchange chromatography on Fractogel or Q-Sepharose. We also optimized enzyme elution methods using ion strength gradient and pH gradient. The enzymes were purified to homogeneity in a single step as confirmed by SDS-PAGE (Supporting Information, Figure S2). The purification yields and hydrolytic activity of recombinant α-l-fucosidases are summarized in Supporting Information, Table S1. *Fg*FCO1 yielded the highest protein amount from 200 mL of cultivation medium (83 mg) but with quite low specific activity of 0.0018 U mg^–1^. *Fo*FCO1 and *Aph*Fuc afforded lower protein yields (2.2 mg and 2.8 mg, respectively) but much higher specific activities (41 and 4.9 U mg^–1^). The purification yields of all proteins were comparable, ca. 13–15%. Both *Fg*FCO1 and *Aph*Fuc showed a rather steep activity decline during purification, with a 4–5-fold higher activity in the cultivation medium compared to the purified form, while *Fo*FCO1 maintained consistent activity across both stages. Therefore, the purification conditions of the prior two enzymes may possibly be optimized to reach higher yields of active protein. The apparent molecular weight of *Aph*Fuc was estimated by size-exclusion chromatography (SEC) to be approximately 190 kDa, based on calibration with standard proteins. This estimate was reproducibly obtained from three independent SEC measurements, which afforded consistent results within the experimental error, indicating that the observed oligomeric state was not artifact. Given that SDS-PAGE analysis indicated a monomeric size of ∼95 kDa (Supporting Information, Figure S2), the SEC result suggests that *Aph*Fuc forms a homodimer under native conditions.

### Biochemical Characterization of Recombinant α-l-Fucosidases

The effect of pH on the activity of recombinant *Fo*FCO1, *Fg*FCO1, and *Aph*Fuc enzymes produced in *P. pastoris* was investigated (Figure 2). The pH optimum was defined as the pH value, at which the highest enzyme activity was achieved. The standard activity assay was performed at the given pH with *p*NP-α-l-Fuc substrate. For *Fo*FCO1, pH optimum was found to be pH 5.5 (specific activity 6.4 U mg^–1^). In the pH range of 5.5–7.5, *Fo*FCO1 retained specific activity higher than 60%. At pH lower than 3.5 and higher than 10.5, *Fo*FCO1 retained less than 5% activity. For *Fg*FCO1, pH optimum was determined in the same way. At pH optimum of 4.5, the enzyme had the highest activity of 0.007 ± 0.002 U mg^–1^. *Fg*FCO1 maintained more than 60% activity at pH 3.0–5.0, but beyond this range its activity steeply decreased to less than 5%. pH optimum for *Aph*Fuc was determined to be 4.0 where the maximum activity of 5.4 U mg^–1^ was reached. *Aph*Fuc maintained more than 60% activity at pH 2.0–4.5; at higher pH its activity decreased. For example, at pH 7.5, the *Aph*Fuc activity was lower than 5%. The temperature optimum of new *Aph*Fuc enzyme was determined to be 65 °C (in a 10 min reaction), with the enzyme being completely deactivated at 85 °C. The temperature optimum for *Fo*FCO1 was determined previously by Yamamoto et al.^43^ to be 50 °C. However, it should be noted that generally, temperature stabilities at prolonged time incubations are much lower in glycosidases. Moreover, increased reaction temperatures strongly shift reaction equilibrium toward hydrolysis over transglycosylation. Therefore, in this work, synthetic reactions were run at 35 °C.

**Figure 2:**
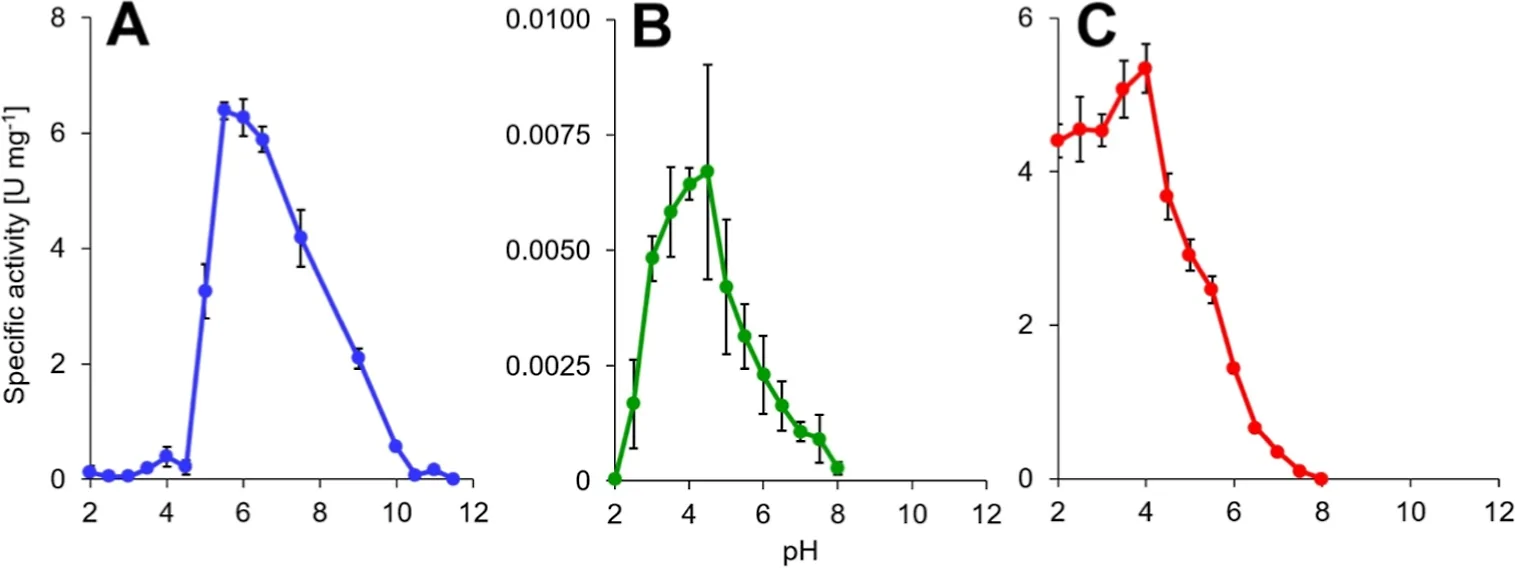
Effect of pH on the hydrolytic activity of purified recombinant α-l-fucosidases. Enzyme activity was measured using *p*NP-α-l-Fuc (2 mM) as a substrate in Britton–Robinson buffer pH 2.0–12.0 at 35 °C as detailed in Materials and Methods. (A), *Fo*FCO1; (B), *Fg*FCO1; (C), *Aph*Fuc.

The determination of kinetic parameters of all three α-l-fucosidases was performed by end-point measurement using substrate *p*NP-α-l-Fuc in the concentration range of ca. 0.5–5 × *K*_M_. The enzyme *Fo*FCO1 was found to have *K*_M_ = 2.4 mmol L^–1^ and *k*_cat_ = 0.3 s^–1^. The previously reported kinetic parameters of native *Fo*FCO1 purified from the fungal medium (*K*_M_ = 0.85 mmol L^–1^, *k*_cat_ = 83 s^–1^) were slightly different, which is probably caused by the different enzyme producer (native—*F. oxysporum*, or heterologous—*P. pastoris*).^16^ Paper et al. also reported on the kinetic parameters of recombinantly produced *Fg*FCO1 (*K*_M_ = 9.8 mmol L^–1^, *k*_cat_ = 510 s^–1^). However, in contrast, our results for *Fg*FCO1 showed a strong allosteric character of the Michaelis–Menten curve, affording *K*_half_ = 4.7 mmol L^–1^; *k*_cat_ could not be reliably determined due to the solubility limitations of the substrate (Table 2 and Supporting Information, Figure S3). The sigmoidal kinetic behavior of recombinant *Fg*FCO1 may be caused by several factors. First, the enzyme used in this work was produced heterologously in *P. pastoris*, causing possible differences in folding, oligomerization state, or post-translational modifications compared to the native enzyme isolated from *Fusarium* culture. Such differences may influence enzyme dynamics and substrate binding behavior in recombinant glycosidases.^44,45^ Second, the observed kinetic behavior may also reflect intrinsic properties of the recombinant enzyme. The artificial substrate *p*NP-α-l-Fuc is a relatively poor substrate for *Fg*FCO1, as the enzyme preferentially acts on natural fucosylated xyloglucan oligosaccharides.^16^ Under such conditions, deviations from ideal Michaelis–Menten behavior may arise because substrate binding and catalytic turnover are not optimally balanced. We hypothesize that our result better correlated with the conclusions by Cao et al.,^20^ who revealed two conformational forms in the crystallographic structure of *Fg*FCO1. According to Cao et al., one of the forms represents the enzyme in a nonreactive state while the other form is catalytically active.^20^ Such conformational dynamics may also have contributed to non-Michaelis–Menten kinetic behavior.

**Table 2:** Kinetic Parameters of Hydrolysis of *p*NP-α-l-Fuc by Recombinant α-l-Fucosidases^a^

α-l-fucosidase	*K*_M_ [mmol L^–1^]	*k*_cat_ [s^–1^]	*k*_cat_/*K*_M_ [L mmol^–1^ s^–1^]	*K*_half_ [mmol L^–1^]
*Fo*FCO1	2.4 ± 0.3	0.30 ± 0.01	0.12	n.a.
*Fg*FCO1	n.a.	n.d.	n.d.	4.7 ± 0.9
*Aph*Fuc	0.30 ± 0.02	5.7 ± 0.1	19	n.a.

a n.a., Not applicable; n.d., not determined.

*Aph*Fuc exhibited markedly different kinetic behavior from *Fo*FCO1 and *Fg*FCO1. Its low *K*_M_ value (0.30 mmol L^–1^) reflected a much higher substrate affinity to *p*NP-α-l-Fuc than *Fo*FCO1 (2.4 mmol L^–1^). Additionally, *Aph*Fuc had a significantly higher turnover rate (*k*_cat_ = 5.7 s^–1^) compared with *Fo*FCO1 (0.30 s^–1^). As a result, the catalytic efficiency of *Aph*Fuc (*k*_cat_/*K*_M_ = 19 L mmol^–1^ s^–1^) was markedly higher than that of *Fo*FCO1 (0.12 L mmol^–1^ s^–1^), indicating superior catalytic performance of *Aph*Fuc with the synthetic substrate *p*NP-α-l-Fuc.

When compared with other characterized fungal GH29 α-l-fucosidases acting on *p*NP-α-l-Fuc, *Aph*Fuc ranked among the most efficient enzymes. Its catalytic efficiency (*k*_cat_/*K*_M_ = 19 L mmol^–1^ s^–1^) was comparable to the highly active *Fp*FucA from *Fusarium proliferatum* (*k*_cat_/*K*_M_ ≈ 36 mM^–1^·s^–1^)^17^ and markedly exceeded that reported for the *A. niger* α-l-fucosidase (*k*_cat_/*K*_M_ ≈ 2.5–5 mM^–1^·s^–1^).^19^ In contrast, *Fo*FCO1 showed low efficiency (0.12 L mmol^–1^ s^–1^), which ranked it at the lower end of the activity spectrum with this synthetic substrate.

The regioselectivity of α-l-fucosidases *Fo*FCO1, *Fg*FCO1, and *Aph*Fuc was investigated in a hydrolytic reaction with two natural substrates, namely 2′-fucosyllactose (2′FL) and 3-fucosyllactose (3FL). The experiment showed that all three enzymes can cleave 2′FL, but are not capable of cleaving 3FL (Figure 3).

**Figure 3:**
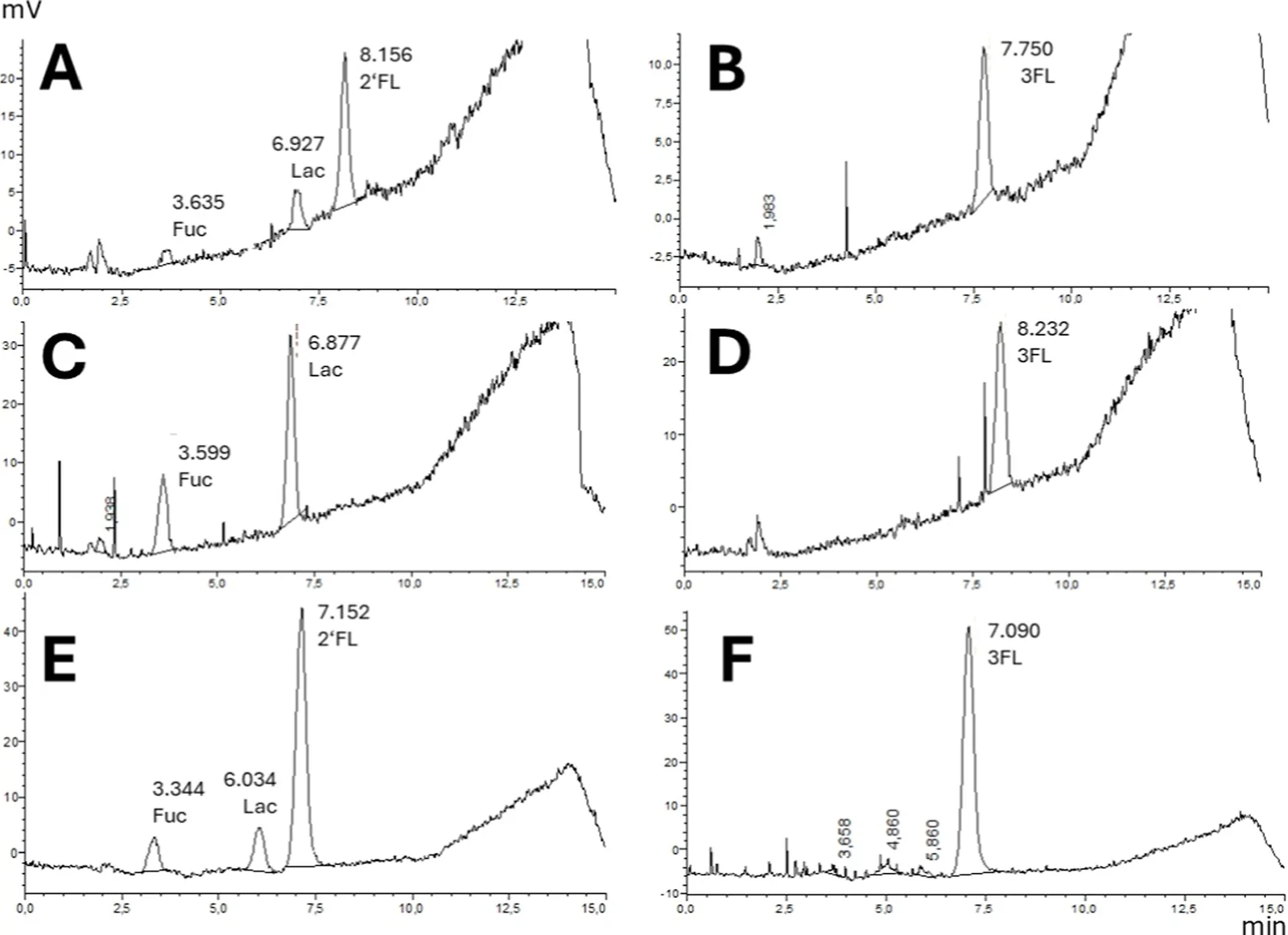
HPLC analysis of hydrolysis of natural substrates 2′-fucosyllactose (2′FL, 24 h) and 3-fucosyllactose (3FL, 48 h) by fungal α-l-fucosidases. (A,B) *Fo*FCO1; (C,D) *Fg*FCO1; (E,F) *Aph*Fuc. Panels A, C, and E show hydrolysis of 2′FL, while panels B, D, and F show hydrolysis of 3FL. Peaks are labeled as follows: Fuc, l-fucose; Lac, lactose; 2′FL, 2′-fucosyllactose; 3FL, 3-fucosyllactose. Retention times were 3.34–3.64 min for fucose, 6.03–6.93 min for lactose, and 7.09–8.23 min for 2′FL/3FL.

### Transfucosylation Capability of Fungal α-l-Fucosidases

As the next step, we investigated the transfucosylation ability of purified α-l-fucosidases. Commercially available *p*NP-α-l-Fuc was used as a donor with various acceptors in a 3-fold molar excess. The acceptors (Figure 4A) comprised monosaccharides: d-galactose (**5**), d-arabinose (**6**), l-fucose (**7**), l-rhamnose (**8**); disaccharides: lactose (**9**), rutinose (**10**); and modified saccharides: lactosyl-*t*Boc (**11**), *p*NP-β-d-galactoside (**12**), *p*NP-β-lactoside (**13**), *p*NP-β-melibioside (**14**). From our experience, the presence of C-1 substitution, especially of hydrophobic/aromatic nature, may sometimes stimulate transglycosylation activity of glycosidases. All the compounds were commercially available, except for lactosyl-*t*Boc (**11**), which was synthesized according to a previously published procedure (Supporting Information, Section S1).^46^ Rutinose (**10**) was prepared as described previously (Supporting Information, Section S1).^47^

**Figure 4:**
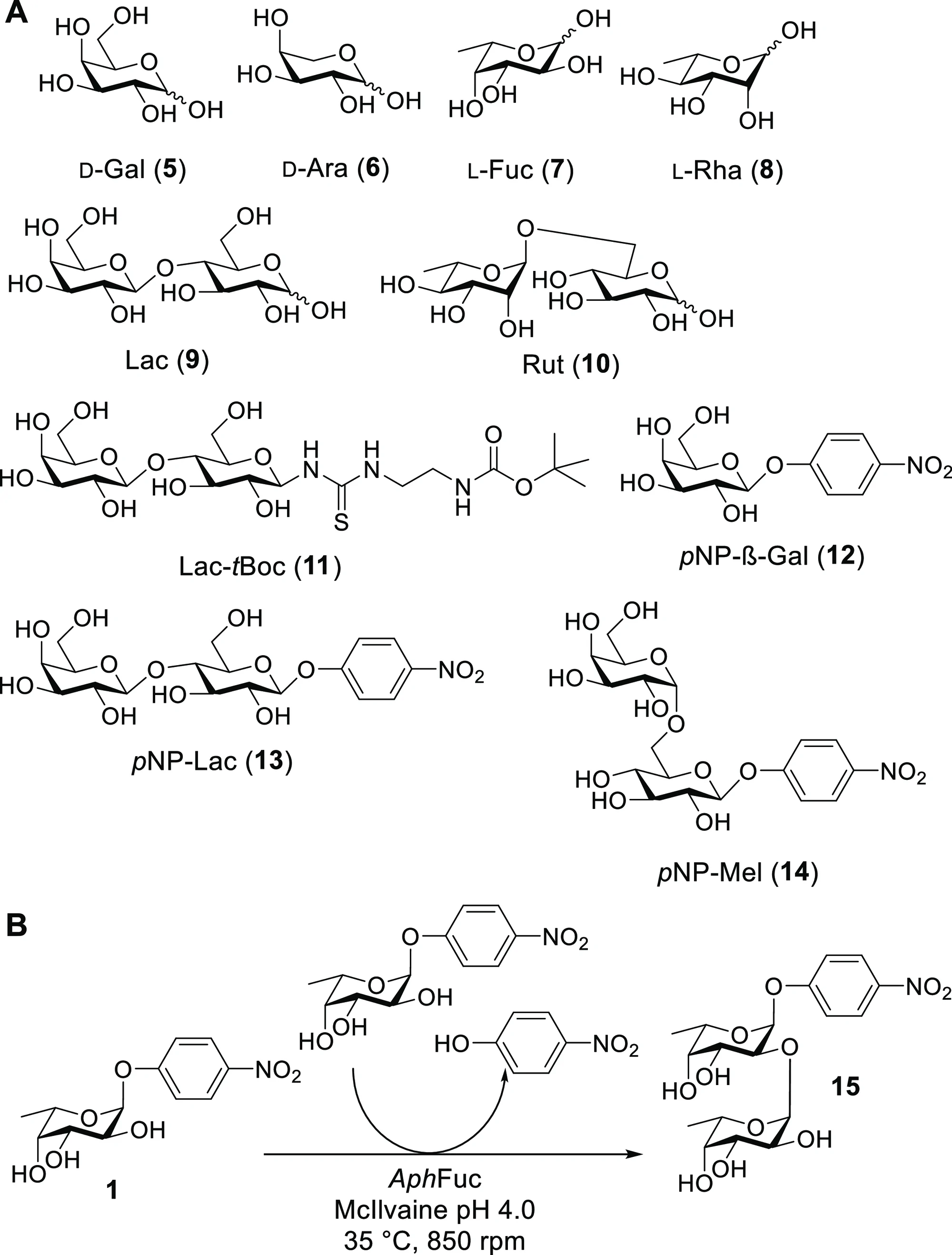
(A) Structures of fucosylation acceptors tested with recombinant α-l-fucosidases: d-galactose (**5**), d-arabinose (**6**), l-fucose (**7**), l-rhamnose (**8**), lactose (**9**), rutinose (**10**), lactosyl-*t*Boc (**11**), *p*NP-β-d-galactoside (**12**), *p*NP-β-lactoside (**13**), *p*NP-β-melibioside (**14**). (B) Enzymatic synthesis of functionalized disaccharide *p*-nitrophenyl α-l-fucopyranosyl-(1 → 2)-α-l-fucopyranoside catalyzed by α-l-fucosidase *Aph*Fuc. To rationalize the acceptor specificity of *Aph*Fuc and the obvious preference of *p*NP-α-l-Fuc (**1**) as an acceptor to other compounds tested, we performed molecular modeling of this enzyme using AlphaFold3. Into the enzyme active site, we docked (i) autocondensation product **15**, and (ii) selected acceptors carrying a *p*NP moiety (**1**, **12**–**14**) along with the glycosyl-enzyme intermediate. The results (Supporting Information, Figures S4–S6) gave more insight into the found transglycosylation behavior as discussed in the next section.

During these analytical reactions we did not observe fucosylation of either of these acceptors, but we clearly observed formation of self-condensation products from *p*NP-α-l-Fuc in the reaction catalyzed by *Aph*Fuc on TLC. While transglycosylation ability of *Fo*FCO1 was never described previously,^16^ the transglycosylation ability of *Fg*FCO1 was previously shown only with xyloglucan from citrus peels as a donor.^41^ Although *Fg*FCO1 exhibited hydrolytic activity with *p*NP-α-l-Fuc substrate, it did not accept it as a donor for transglycosylation, which corresponds to our results.

For the preparative autocondensation reaction, product isolation and characterization, we selected the yet undescribed enzyme *Aph*Fuc. In this reaction, *p*NP-α-l-Fuc acted both as a fucosyl donor and an acceptor (Figure 4B). The autocondensation reaction led to a single product *p*-nitrophenyl α-l-fucopyranosyl-(1 → 2)-α-l-fucopyranoside (**15**) in a 20% isolated yield. The found α-(1 → 2)-regioselectivity was in accord with the enzyme hydrolytic activity with 2′-fucosyllactose. For structural characterization, see Supporting Information, Table S2 and Figures S11–S14.

## Discussion

The present study aimed to identify novel fungal α-l-fucosidases of the GH29 family suitable for scalable preparative fucosylations. By combining induction-based screening of fungal producers, in silico sequence analysis, heterologous expression, and detailed biochemical and synthetic characterization, we discovered, heterologously expressed, and characterized a new fungal α-l-fucosidase, *Aph*Fuc, as a candidate with a notable transfucosylation potential.

*Aph*Fuc displayed distinct biochemical properties including a high catalytic efficiency and an acidic pH optimum (pH 4.0) useful for specific industrial and biotechnological applications. The apparent molecular weight of *Aph*Fuc, estimated to be approximately 190 kDa by size-exclusion chromatography (SEC), suggests a dimeric organization under native conditions. This dimeric state is notable, as GH29 α-l-fucosidases have so far been predominantly reported as monomeric enzymes.^29,48,49^ *Fg*FCO1 was shown to form a monomeric structure in solution, as confirmed by SAXS despite the presence of two molecules in the crystallographic asymmetric unit (PDB: 4NI3).^20^ Homologous α-l-fucosidase from *F. proliferatum* was also crystallized as a monomer (PDB: 9LXL), with no evidence of oligomerization reported.^21^ Similarly, *Fo*FCO1 was previously estimated to have a molecular weight of ∼75 kDa by gel filtration, consistent with a monomeric form.^43^ The observed behavior may therefore represent an unusual structural feature within this enzyme class.

Transfucosylation activity within the GH29 family has been predominantly reported for bacterial enzymes,^26,41,50,51^ whereas fungal representatives are exceptionally rare and often display strictly hydrolytic behavior. In this context, the identification and characterization of *Aph*Fuc as a novel fungal α-l-fucosidase capable of efficient regioselective α-(1 → 2)-transfucosylation with a priceworthy synthetic substrate *p*NP-α-l-Fuc under mild aqueous conditions represents a valuable addition. In the family of fungal GH29 α-l-fucosidases, *Fg*FCO1 catalyzed transfucosylation affording a mixture of 2′-fucosyllactose together with another regioisomer, using α2-fucosylated xyloglucan nonasaccharide as a donor.^41^ α-l-Fucosidase from *A. niger* used *p*NP-α-l-Fuc as a donor for transglycosylation, but in the reaction with lactose it formed nonreducing 1-fucosyllactose as a only product.^19^ The α-(1 → 2) regioselectivity of *Aph*Fuc may be particularly relevant for the synthesis of human milk oligosaccharides (HMOs) where the α-(1 → 2)-fucosylated motif is the key structural element.^52,53^ Although lactose did not act as an acceptor in the present study, the demonstrated regioselectivity of *Aph*Fuc suggests potential for future engineering aimed at expanding its acceptor scope toward lactose and HMOs.

The strict acceptor specificity of *Aph*Fuc despite the large panel of tested potential acceptors may indicate structural and mechanistic constraints of alternative acceptors in the enzyme active site, restricting productive binding modes required for glycosyl transfer.^54,55^ In addition, the aromatic *p*-nitrophenyl moiety of *p*NP-α-l-Fuc may have enhanced acceptor binding through favorable hydrophobic and π–π interactions, thereby promoting autocondensation. Similar limitations in acceptor scope have been reported for other GH29 α-l-fucosidases, where efficient transglycosylation was often restricted to structurally optimized substrates or required high donor excess, organic cosolvents, or reduced water activity.^41,56,57^ Recent studies have shown that targeted enzyme engineering, particularly mutagenesis of residues shaping the +1 and +2 subsites, can substantially broaden acceptor tolerance and improve synthetic performance.^55,58,59^ This mutagenesis strategy may also be applicable to *Aph*Fuc, expanding its substrate scope toward natural acceptors such as lactose, and opening new routes for the synthesis of biologically relevant fucosylated oligosaccharides, including HMO analogues.

The strict acceptor specificity observed for *Aph*Fuc apparently reflects the structural constraints within its acceptor-binding region. In GH29 α-l-fucosidases, the +1 subsite responsible for acceptor binding is often relatively shape-selective, allowing productive positioning only for substrates with a precise orientation of nucleophilic hydroxyl groups.^20,41^ Based on the preference of *Aph*Fuc for 2′-fucosyllactose in contrast to 3-fucosyllactose, we hypothesize that the C-2 hydroxyl of the galactose-capped acceptor must be in the correct orientation for a nucleophilic attack.^20^ Bulky or structurally distinct acceptors, including alternative mono- and disaccharides tested in this study, may therefore be unable to adopt the productive binding mode within the active site. In glycosidases, hydrolysis strongly competes with glycosyl transfer in aqueous medium, and so a productive acceptor binding must occur within a limited time window during the lifetime of the glycosyl-enzyme intermediate. If the acceptor is not rapidly and precisely positioned, donor hydrolysis becomes the dominant reaction pathway.

Molecular modeling of *Aph*Fuc with the autocondensation product **15** (Supporting Information, Figures S4 and S5) indicated that the aglycone-binding region is partially shielded from solvent and enriched in aromatic residues, forming a favorable environment for binding of the *p*-nitrophenyl group, which may stabilize the acceptor in the productive orientation by hydrophobic and π–π interactions. In contrast, smaller acceptors such as free fucose, lacking these stabilizing interactions, bind less efficiently, affording no transglycosylation products. The specific residue composition of the aglycone-binding site thus appears to restrict the range of acceptable acceptors.

The docking analysis of the acceptors carrying the *p*NP moiety (Supporting Information, Figure S6) showed that all tested acceptors (*p*NP-α-l-Fuc **1**, *p*NP-β-d-galactoside **12**, *p*NP-lactoside **13**, and *p*NP-melibioside **14**) were accommodated within the +1/+2 subsites with comparable binding scores. Whereas *p*NP-lactoside (**13**), and *p*NP-melibioside (**14**) could be docked with their C-3 hydroxyl close to the catalytic residue, it was C-2 hydroxyl in *p*NP-β-d-galactoside (**12**) and *p*NP-α-l-Fuc (**1**). The distance between the nucleophilic hydroxyl group and the anomeric carbon differed significantly between acceptors, from ∼0.33 nm for **1** to ∼0.37–0.48 nm for **12**–**14**. Since productive transglycosylation requires precise positioning of the acceptor hydroxyl group relative to both the catalytic acid/base residue and the anomeric carbon of the intermediate,^60^ these differences probably caused the transglycosylation inefficiency with acceptors other than **1**. Presumably, the presence of the *p*NP aglycone must combine with the stereochemical compatibility of the acceptor carbohydrate moiety to reach productive positioning.

In conclusion, this work identified a novel fucosylation tool, fungal α-l-fucosidase *Aph*Fuc. The fucosylation activity of this enzyme, although limited by its narrow acceptor scope, is exceptional within the family of recombinant fungal α-l-fucosidases, among which synthetic activities are rare. Its regioselectivity, stability across a broad pH range, and efficient transfucosylation capability provide a strong foundation for its application in the synthesis of fucosylated products. Future work may focus on its engineering to enhance its transglycosylation efficiency and broaden its substrate scope toward other acceptors besides autocondensation.

## Supplementary Material



## Notes

Materials (Section S1); analytical methods (Section S2); synthetic methods (Section S3); production and characterization of α-l-fucosidases (Section S4); molecular modeling of *Aph*Fuc and docking analysis (Section S5); structural characterization of prepared compounds (Section S6). These sections contain the following items: synthesis of quinovose (Scheme S1); dependence of extracellular α-l-fucosidase activity on the day of cultivation (Figure S1); purification of produced recombinant α-l-fucosidases (Table S1); SDS-PAGE analysis of recombinant α-l-fucosidases (Figure S2); Michaelis–Menten kinetic curves of hydrolysis of *p*NP-α-l-Fuc by recombinant α-l-fucosidases (Figure S3); hydrogen bonding interactions of product and *Aph*Fuc (Figure S4); *Aph*Fuc surface with product (Figure S5); interactions of selected transglycosylation acceptors (Figure S6); characterization of prepared quinovose by ^1^H and ^13^C NMR, MS and HPLC (Figures S7–S10); characterization of prepared *p*-nitrophenyl α-l-fucopyranosyl-(1 → 2)-α-l-fucopyranoside by ^1^H and ^13^C NMR, MS and HPLC (Table S2 and Figures S11–S14) (PDF)

## Abbreviations

2′FL: 2′-fucosyllactose
3FL: 3-fucosyllactose
*Ap*Fuc: α-l-fucosidase from *Apiotrichum porosum*
*Aph*Fuc: α-l-fucosidase from *Aspergillus phoenicis*
*Ar*Fuc: α-l-fucosidase from *Absidia repens*
CNP-α-l-Fuc: 2-chloro-4-nitrophenyl α-l-fucopyranoside
CAZy: Carbohydrate Active enZymes database
CCF: Culture Collection of Fungi
*Cr*Fuc: α-l-fucosidase from *Clonostachys rhizophaga*
*Fg*FCO1: α-l-fucosidase from *Fusarium graminearum*
*Fo*FCO1: α-l-fucosidase from *Fusarium oxysporum*
GH: glycoside hydrolase
HMO: human milk oligosaccharide
*p*NP-α-l-Fuc: 4-nitrophenyl α-l-fucopyranoside
SEC: size-exclusion chromatography
*Tf*Fuc: α-l-fucosidase from *Penicillium funiculosum*
*Tp*Fuc: α-l-fucosidase from *Penicillium purpurogenum*
*Tm*αFuc: α-l-fucosidase from *Thermotoga maritima*
