# Supplementary material for: Identification of a Novel Fungal α‑l‑Fucosidase with Transfucosylation Potential
Source: ACS Omega. 2026 Aug 4;11(32):47335–45. doi: 10.1021/acsomega.6c00254 (PMC13491509; doi:10.1021/acsomega.6c00254)
Supplement: Supplementary file 1 [file ao6c00254_si_001.pdf]

Supporting Information

Identification of a Novel Fungal α-L-Fucosidase With Transfucosylation Potential

Pavlína Nekvasilová[1, 2]; Michaela Glozlová[1]; Andrea Vopálenská[1, 3]; Lucie Petrásková[1]; Natalia Kulik[1]; Pavla Bojarová[1*]

[1] Laboratory of Biotransformation, Institute of Microbiology, Czech Academy of Sciences, Vídeňská 1083, CZ-14200 Prague 4, Czech Republic

[2] Department of Genetics and Microbiology, Faculty of Science, Charles University, Viničná 5, CZ-12843 Prague 2, Czech Republic

[3] Department of Organic Chemistry, Faculty of Science, Charles University, Hlavova 2030/8, CZ-12800 Prague 2, Czech Republic

Correspondence: Pavla Bojarová (bojarova@biomed.cas.cz)

Content

1. Materials
2. Analytical Methods
3. Synthetic Methods
4. Production and Characterization of α-L-Fucosidases
5. Molecular Modeling of *Aph*Fuc and Docking Analysis
6. Structural Characterization of Prepared Compounds
7. References

1. Materials

Galactose and rhamnose were obtained from Sigma-Aldrich (USA). *p*NP-α-L-fucopyranoside (**1**; 4-nitrophenyl-α-L-fucopyranoside), *p*NP-β-D-galactopyranoside (**12**; 4-nitrophenyl-β-D-galactopyranoside), *p*NP-β-lactoside (**13**; 4-nitrophenyl β-D-galactopyranosyl-(1→4)-β-D-glucopyranoside), *p*NP-β-melibioside (**14**; 4-nitrophenyl α-D-galactopyranosyl-(1→6)-β-D-glucopyranoside), D-arabinose, and L-fucose were purchased from Biosynth (UK). Restriction enzymes were purchased from New England Biolabs (USA).

Rutinose (**10**; α-L-rhamnopyranosyl-(1→6)-D-glucopyranose) was prepared according to Kapešová et al. (2019)[1] by hydrolysis of rutin (Alchimica, CZ) using recombinant rutinosidase from *Aspergillus niger*. The reaction was carried out under "solid-state biocatalysis" conditions, where both the substrate (rutin) and the hydrolytic product (quercetin) remained largely undissolved in suspension. This setup enabled efficient conversion without the need of co-solvents or toxic reagents. As quercetin precipitated from the reaction mixture, rutinose remained in the aqueous phase and was then isolated from the filtrate using gel permeation chromatography on a Biogel P-2 column with water as a mobile phase in 63.0% yield.[1-2]

Lactosyl-*t*Boc (**11**; Lac-*t*Boc; (t-butoxycarbonylamino)ethylthioureidyl β-D-galactopyranosyl-(1→4)-β-D-glucopyranoside) was prepared as described previously[3] in a five-step synthetic procedure starting from lactose. Lactose was first per-*O*-acetylated by acetic anhydride affording per-*O*-acetylated lactose. The next step was regioselective bromination of per-*O*-acetylated lactose in the presence of hydrobromic acid to form glycosyl bromide, which was directly used in the subsequent reaction with potassium thiocyanate and a phase transfer catalyst (tetrabutylammonium hydrogensulfate), affording the isothiocyanate product. The latter was then allowed to react with *N*-Boc-ethylenediamine to form the thiourea product, which was finally deprotected under conditions by Zemplén, leading to lactosyl-*t*Boc in an overall yield of 35%.

6-Deoxy-D-glucose (**4**; quinovose) was prepared in five steps from methyl α-D-glucopyranoside (**1**; Scheme S1). The primary hydroxyl of glycoside **1** was first converted to iodide in the presence of triphenylphosphine and its free hydroxyl groups were acetylated. Subsequent catalytic dehalogenation of iodide **2** resulted in dehalogenated product **3**. Acetolysis of **3** using sulfuric acid and acetic anhydride followed by Zemplén deacetylation resulted in 6-deoxy-D-glucose **4** in an overall yield of 23%. For a detailed description of the synthesis, see Section 3. For the structural characterization of compound **4** see Figures S7-S10.

Unless otherwise stated, all other chemicals, such as inorganic salts, medium components, organic solvents, were purchased from VWR Chemicals (CZ).

2. Analytical Methods

2.1. HPLC Analysis

HPLC analysis was used to monitor the course of transglycosylation reactions. The Shimadzu Prominence LC analytical system was comprised of a Shimadzu CBM-20A system controller, Shimadzu CTO-10AS column oven, Shimadzu LC-20AD binary HPLC pump, Shimadzu SIL-20ACHT cooling autosampler, Shimadzu SPD-20MA diode array detector (Shimadzu, Japan), and PL 1000 evaporative light scattering detector (Polymer Laboratories, USA). Analyses were performed on a Luna NH_2 column (150 × 4.6 mm, 5 μm) preceded by security guard NH_2 cartridge (4 × 3 mm, Phenomenex, USA) in acetonitrile/ water (4/1, *v*/*v*), with gradient elution as follows (A = acetonitrile, B = water): 21% B for 0 min, 21-30% B for 0-8 min, 30-50% B for 8-12 min, 50-21% B for 12-13 min and 21% B for 2 min for column equilibration; flow rate of 1.1 mL min^{-1} at 25 °C; injection volume 1 μL. Detection was performed at 200 nm, sampling 40 Hz. Operation parameters for evaporative light scattering detector were as follows: gas (N_2) flow 0.8 mL min^{-1}, nebulizer temperature 40 °C, evaporator temperature 85 °C, sampling 2 Hz.

2.2. Mass Spectrometry Analysis

High-resolution mass spectrometry analysis was performed using LTQ Orbitrap XL hybrid mass spectrometer (Thermo Fisher Scientific, USA) equipped with an electrospray ion source. The mobile phase was methanol/water (4:1, *v*/*v*) at a flow rate of 100 μL min^{-1}. The samples were dissolved in methanol or methanol/water and injected using a 5-μL loop into the mobile phase flow. In the positive ion mode, spray voltage, capillary voltage, tube lens voltage, and capillary temperature were 5.0 kV, 9 V, 150 V, and 275 °C, respectively. The spectra were recorded at a resolution of 100,000.

2.3. NMR Analysis

NMR spectra were recorded on a Bruker Avance III 700 MHz (compound **4**) or a Bruker Avance III 600 MHz (compound **15**) spectrometer (Bruker BioSpin, DE) in D_2O (99.90 atom % D, VWR Chemicals, BE) at 30 °C. The residual signal of D_2O (δ_H 4.732 ppm) was used as the proton spectra reference; carbon spectra of compounds **4** and **15** were referenced to the signal of methanol (δ_C 49.05 ppm) and acetone (δ_C 30.50 ppm), respectively. Compound **4** was assigned using COSY and HSQC experiments. The individual monosaccharide units of compound **15** were assigned using COSY, HSQC, and 1D-TOCSY experiments performed using manufacturer's software; their anomeric configuration was deduced from the magnitude of J(H1,H2) coupling constant. Glycosidic linkage was confirmed by the HMBC correlation between the involved carbon and the adjacent anomeric proton.

3. Synthetic Methods

3.1. Synthesis of 6-Deoxy-D-glucopyranose (quinovose; 4)

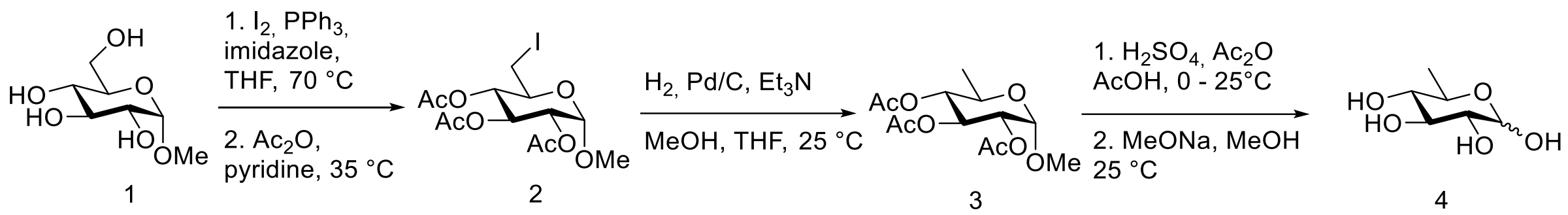

Scheme S1. Synthesis of quinovose (**4**).

Methyl 2,3,4-tri-*O*-Acetyl-6-iodo-α-D-glucopyranoside (2)

Triphenylphosphine (8.7 g, 33.5 mmol, 1.3 equiv) and imidazole (2.8 g, 41.2 mmol, 1.6 equiv) were added to a stirred solution of methyl α-D-glucopyranoside (**1**; 5.0 g, 25.8 mmol, 1 equiv) in tetrahydrofuran (25 mL) at room temperature. Then, solution of iodine (8.5 g, 33.5 mmol, 1.3 equiv) in tetrahydrofuran (13 mL) was dropwise added to the reaction mixture over 2 h at 70 °C. Then, the reaction mixture was stirred at 70 °C for 2 h. After complete consumption of starting glucoside **1** (monitored by TLC, DCM/MeOH = 4/1, *v/v*), the reaction mixture was cooled down to room temperature, followed by the addition of pyridine (13 mL) and acetic anhydride (12 mL, 128.8 mmol, 5 equiv). The resulting reaction mixture was stirred at 35 °C overnight. With complete consumption of intermediate as indicated by TLC (DCM/MeOH = 4/1, *v/v*, cyclohex/EtOAc = 1/1, *v/v*), the reaction mixture was cooled down to 0 °C and carefully quenched (slightly exothermic reaction) with methanol (25 mL). Then, the mixture was diluted with EtOAc (30 mL), washed with water (40 mL), and the water phase was extracted with EtOAc (3 × 10 mL). The organic extracts were collected and washed with 5 M H_2SO_4 (2 × 60 mL), water (2 × 60 mL) and 0.2 M aqueous sodium thiosulfate (2 × 60 mL). The organic phase was dried over Na_2SO_4. After the drying agent was filtered off, solvents were removed under reduced pressure. The crude product was purified by column chromatography (cyclohex/EtOAc = 3/1, *v/v*), followed by crystallization from boiling EtOH (15 mL), affording acetate **2** (8.2 g, 75% yield) as a white crystalline solid. The NMR spectra were identical to the previously reported data.[4]

Methyl 2,3,4-tri-*O*-Acetyl-6-deoxy-α-D-glucopyranoside (3)

Triethyl amine (9.3 mL, 67.2 mmol, 3 equiv) was added to the solution of acetylated iodide **2** (9.6 g, 22.4 mmol, 1 equiv) in tetrahydrofuran (40 mL) and MeOH (80 mL) at room temperature. Then, palladium on carbon (10% *w/w*, 4.8 g, 4.48 mmol, 0.2 equiv) was added under argon atmosphere and the suspension was purged with hydrogen. The reaction mixture was stirred under hydrogen atmosphere at room temperature for 2 h until the complete consumption of acetylated iodide **2** was indicated by TLC (cyclohex/EtOAc = 1/1, *v/v*). Then, the reaction mixture was filtered through celite, washed with MeOH, and the filtrate was concentrated under reduced pressure. The oily residue was dissolved in DCM (50 mL) and washed with water (50 mL). The water phase was extracted with DCM (3 × 10 mL). The organic extracts were collected and washed with 1 M hydrochloric acid (2 × 50 mL) and brine

(1 × 50 mL). The organic phase was dried over Na_2SO_4. After the drying agent was filtered off, the solvents were removed under reduced pressure, affording product **3** (6.4 g, 95% yield) as a pale orange oil. The NMR spectra were identical to the published ones and were in accord with the compound structure.[5]

6-Deoxy-D-glucopyranose (4)

Concentrated sulfuric acid (2.3 mL, 42.3 mmol, 2 equiv) was added dropwise to the solution of glycoside **3** (6.4 g, 21.2 mmol, 1 equiv) in acetic anhydride (100 mL) at 0 °C. The reaction mixture was heated to room temperature and left stirring for 1 h. After the complete consumption of glycoside **3** as indicated by TLC (cyclohex/EtOAc = 3/1, *v/v*) the reaction mixture was cooled to 0 °C and quenched with methanol (150 mL). The reaction mixture was concentrated, the residue was dissolved in EtOAc (50 mL), washed with water (50 mL), and the water phase was extracted with EtOAc (3 × 10 mL). The combined organic extracts were collected and washed with saturated aqueous solution of $NaHCO_3$ (2 × 50 mL) and brine (1 × 50 mL). The organic phase was dried over Na_2SO_4. After the drying agent was filtered off, the solvents were removed under reduced pressure. The resulting crude product was purified by column chromatography (cyclohex/EtOAc = 3/1, *v/v*), affording acetate intermediate (2.4 g, 34% yield), which was directly subjected to deacetylation by Zemplén. A freshly prepared solution of 0.2 M MeONa (0.3 mL) in anhydrous MeOH was dropwise added to a stirred solution of acetate intermediate (7.3 mmol) in anhydrous MeOH (15 mL) to reach pH~8 at room temperature. The reaction mixture was stirred for 1 h at room temperature. After the complete consumption of acetate intermediate as indicated by TLC (cyclohex/EtOAc = 3/1, *v/v,* DCM/MeOH = 3/1, *v/v*), the reaction mixture was neutralized with Dowex 50W H^+ ion exchange resin. After the resin was filtered off and washed with MeOH, the filtrate was concentrated under reduced pressure. Freeze-drying of the resulting oily residue afforded title product **5** (1.1 g, 32% yield) as a colorless oil. NMR spectroscopy analysis revealed a mixture of anomers (α/β = 1/2.4). The NMR spectra were identical to the published ones and were in accord with the compound structure.[6] For structural characterization, see Figures S4-S7.

3.2. Synthesis of *p*-Nitrophenyl α-L-Fucopyranosyl-(1→2)-α-L-fucopyranoside (15)

*p*NP-α-L-Fuc (**5**, 171 mg, 600 µmol) and *Aph*Fuc (6 µL, 0.6 U, 77.4 µg) were incubated in McIlvaine buffer pH 4.0 (total reaction volume 6 mL) at 35 °C and 850 rpm. The reaction progress was monitored by TLC (*i*PrOH/H_2O/NH_4OH *aq*., 7/2/1, *v/v/v*) and by HPLC. The reaction ran for 24 h and was stopped by boiling for 5 min. The reaction mixture was centrifuged (13,500 rpm, 10 min) and the product was purified by gel permeation chromatography on Biogel P-2 column (26×1000 mm, Bio-Rad, UK) with water as a mobile phase at an elution rate of 6.5 mL h^{-1} and ambient temperature. Fractions containing the products were combined and lyophilized. The traces of *p*-nitrophenol were removed by extraction into ethyl acetate. The title compound **15** was obtained as a yellowish powder in the amount of 32.5 mg (98.2 µmol, 20% isolated yield). Its structure was confirmed by NMR and its purity was confirmed by HRMS and HPLC (see Table S2 and Figures S8-S11).

4. Production and Characterization of α-L-Fucosidases

4.1. Production and Purification of Recombinant α-L-Fucosidases in *Pichia Pastoris*

Designed genes (see Sections 3.1 and 2 of the main text) *tpfuc*, *tffuc*, *aphfuc*, *apfuc*, *arfuc*, and *crfuc* were cloned into the pPICZαA vector using *Not*I/*Xba*I (*tpfuc*), *Eco*RI/*Xba*I (*tffuc*), *Eco*RI/*Kpn*I (*aphfuc* and *apfuc*) or *Eco*RI/*Xho*I (*arfuc* or *crfuc*) restriction sites, downstream of the α-factor that provides extracellular targeting of the enzyme and resistance to zeocin.

The plasmids carrying the genes encoding for *Ap*Fuc, *Ar*Fuc, *Cr*Fuc*, Tp*Fuc*, Tf*Fuc *and Aph*Fuc enzymes were linearized (20 µg) using restriction endonuclease *Sac*I or *Pme*I and then electroporated into *Pichia pastoris* KM71H competent cells. For all manipulation with *P. pastoris* we followed the standard protocols by Invitrogen (EasySelect Pichia Expression Kit; Invitrogen, Life Technologies, USA).

First, the screening of the protein expression and enzyme activity on a small scale was performed in a combination of BMGY medium (Buffered Glycerol complex Medium) and BMMY medium (Buffered Methanol complex Medium). Colonies selected after electroporation were inoculated into BMGY medium (100 mL) and incubated at 28 °C and 220 rpm overnight. On day 2, the cultures were centrifuged (5000 rpm, 12 °C, 10 min), and the biomass was transferred into BMMY medium (30 mL) and resuspended. The expression of recombinant α-L-fucosidases was induced by methanol (0.5% *v/v*). Cultures were grown under the same conditions for three days and induced by methanol (0.5% *v/v*) daily. On day 5, the cultures were centrifuged, and the supernatants were tested for α-L-fucosidase expression by SDS-PAGE and standard activity assay (Section 4.2). Positive clones showing expression of α-L-fucosidase were cryopreserved for long-term storage at −80 °C in 15% *v/v* glycerol.

Preparative-scale productions of *Fg*FCO1, *Fo*FCO1 and *Aph*Fuc were performed in a combination of BMGH medium (Buffered Glycerol Minimal Medium) and BMMH medium (Buffered Methanol Minimal Medium). First, YPD medium (yeast extract 10 g L^{-1}, peptone 20 g L^{-1}, D-glucose 20 g L^{-1}; 15 mL) was inoculated by the cryopreserved culture (100 µL) and incubated at 28 °C and 220 rpm for approximately 5 h. Then, BMGH medium (1 L; 3 L Erlenmeyer flask) was inoculated by the preculture and incubated under the same conditions overnight. On day 2, the culture was centrifuged (5000 rpm, 10 min, 12 °C) and the pellet was resuspended in BMMH medium (200 mL; 1 L Erlenmeyer flask). The expression was started by the addition of methanol (0.5% *v/v*). The culture was incubated under the same conditions as the small-scale production and the induction was repeated every 24 h by the addition of methanol (0.5% *v/v*). On day 5, the culture was centrifuged (5000 rpm, 10 min, 12 °C) and the supernatant was loaded onto an ion exchange chromatography column connected to an Äkta Purifier system (Amersham Biosciences, Sweden). *Aph*Fuc and *Fg*FCO1 were purified by cation exchange chromatography (Fractogel EMD SO^{3-}, Merck, DE) on a column equilibrated with 10 mM sodium citrate-phosphate buffer pH 3.5. *Aph*Fuc elution was performed by a linear gradient of 0-1 M NaCl (60 mL) and the elution of *Fg*FCO1 was performed by a linear gradient of 0-100 mM NaOH (60 mL).

Purification of *Fo*FCO1 was performed by anion exchange chromatography (Q-Sepharose, GE Healthcare, SW) on a column equilibrated with 10 mM sodium citrate-phosphate buffer pH 6.5 and eluted by a linear gradient of 0-1 M NaCl (60 mL). The enzymes were purified to homogeneity, and their purity was verified by SDS-PAGE. The enzyme solutions were concentrated by ultrafiltration in McIlvaine buffer (50 mM citric acid/100 mM Na_2HPO_4, pH 4.0), and stored at 4 °C.

4.2. Protein Characterization and Standard Assay for Enzyme Activity

The purity of enzymes was verified by SDS-PAGE in 10% polyacrylamide gel and compared with Low Molecular Weight marker (Cytiva, US). The protein concentration was determined by Bradford method[7] using Protein Assay Dye Reagent Concentrate (Bio-Rad, UK) calibrated for bovine plasma γ-globulin (Bio-Rad, UK).

The apparent molecular weight of *Aph*Fuc was estimated by size-exclusion chromatography using a Superdex 200 10/300 GL column (Amersham Biosciences, GB) in three independent size-exclusion chromatography (SEC) measurements. The column was equilibrated with 10 mM sodium citrate-phosphate buffer, pH 4.0, containing 150 mM NaCl, and operated at a flow rate of 0.3 mL min^{-1}. Protein standards of known molecular weights (i.e., blue dextran, apoferitrin, albumin, alcoholdehydrogenase, and carbonic anhydrase; Sigma-Aldrich, US) were used to calibrate the column. Elution volumes were plotted against the logarithm of molecular weight to generate a standard curve, from which the molecular weight of *Aph*Fuc was interpolated.

The hydrolysis of natural fucosylated compounds 2'-fucosyllactose (2'FL) and 3-fucosyllactose (3FL) was performed analogously. The reaction mixture of 2 mM substrate (2'FL or 3FL), McIlvaine buffer pH 5.0, and an appropriately diluted enzyme was incubated at 35 °C at 850 rpm for 24 h. The reaction was then stopped by boiling at 99 °C for 5 minutes and analyzed by HPLC (see Section 2.1).

The pH optimum of α-L-fucosidases was determined in the universal Britton-Robinson buffer (0.04 M H_3PO_4/0.04 M phenylacetic acid/0.04 M H_3BO_3/0.2 M NaOH) pH 2 – 12 under the conditions of the standard activity assay as above.

Michaelis-Menten parameters of hydrolysis of *p*NP-α-L-Fuc were measured in an end-point kinetic assay. The reaction mixture (400 μL) containing the substrate, McIlvaine buffer pH 5.0 and an appropriately diluted α-L-fucosidase was incubated at 35 °C and 850 rpm for 7 min. Aliquots (50 μL) were taken every 1 min and transferred into 96-well plates containing 1 M sodium carbonate (100 μL) to stop the reaction. Then, absorbance at 420 nm was determined by Sunrise Microplate Reader (Tecan, CHE). The kinetic parameters were extracted by non-linear regression using GraphPad Prism 9 (GraphPad Software, USA).

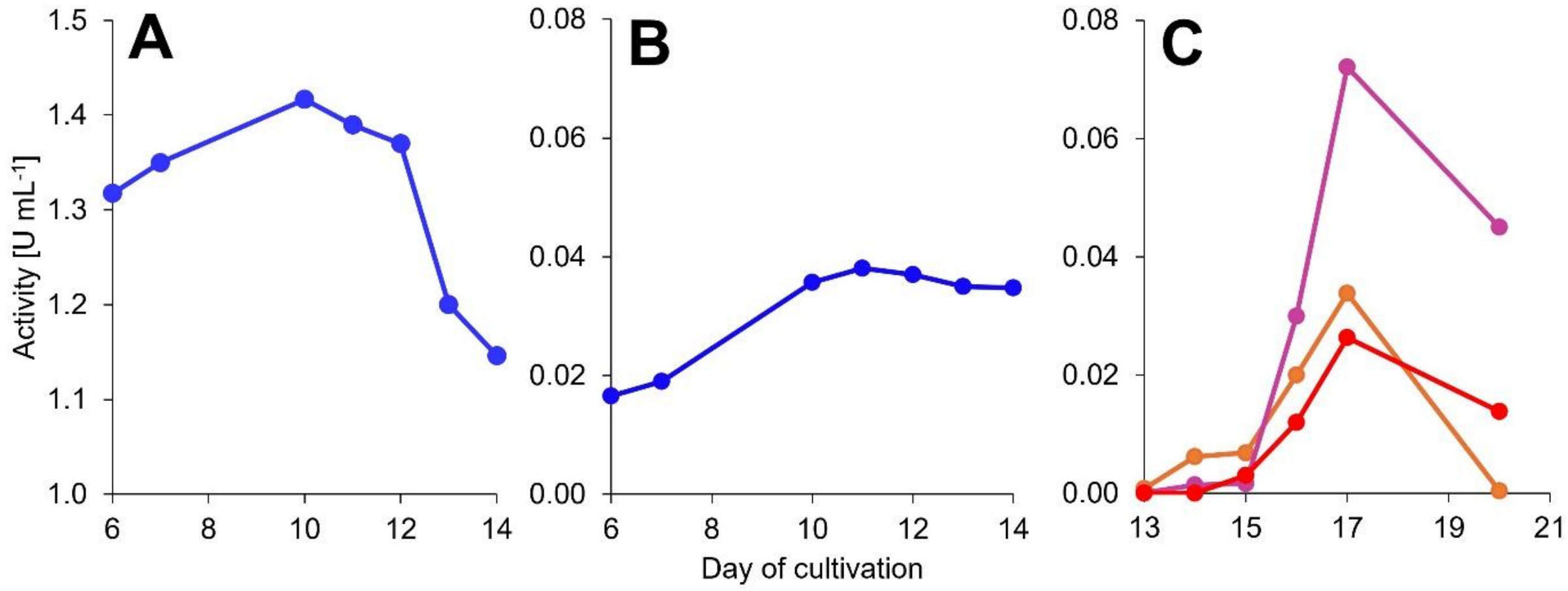

Figure S1. Time course of extracellular α-L-fucosidase activity during cultivation of fungal strains in the presence of inducers. Cultivations were performed in peptone medium at 28 °C with shaking at 220 rpm. α-L-Fucosidase activity was determined in the cultivation supernatant using *p*NP-α-L-Fuc as a substrate: **A.** *F. oxysporum* induced by L-fucose, **B.** *F. oxysporum* induced by D-arabinose, **C.** *P. purpurogenum* (orange), *A. phoenicis* (red), and *P. funiculosum* (fuchsia), all induced by quinovose.

Table S1. Purification (from 200 mL of cultivation medium) of produced recombinant α-L-fucosidases, and their specific activities.

α-L-fucosidase	Yield [mg]		Specific activity [U mg^{-1}]		Purification yield [%]
	Cultivation medium	Purification, ultrafiltration	Cultivation medium	Purification, ultrafiltration	
*Fg*FCO1	117 ± 5	83 ± 2	(10 ± 1).10^{-3}	(1.8 ± 0.4).10^{-3}	13
*Fo*FCO1	14.8 ± 0.3	2.2 ± 0.1	41 ± 3	41 ± 3	14
*Aph*Fuc	18.8 ± 0.8	2.8 ± 0.2	22 ± 1	4.9 ± 0.3	15

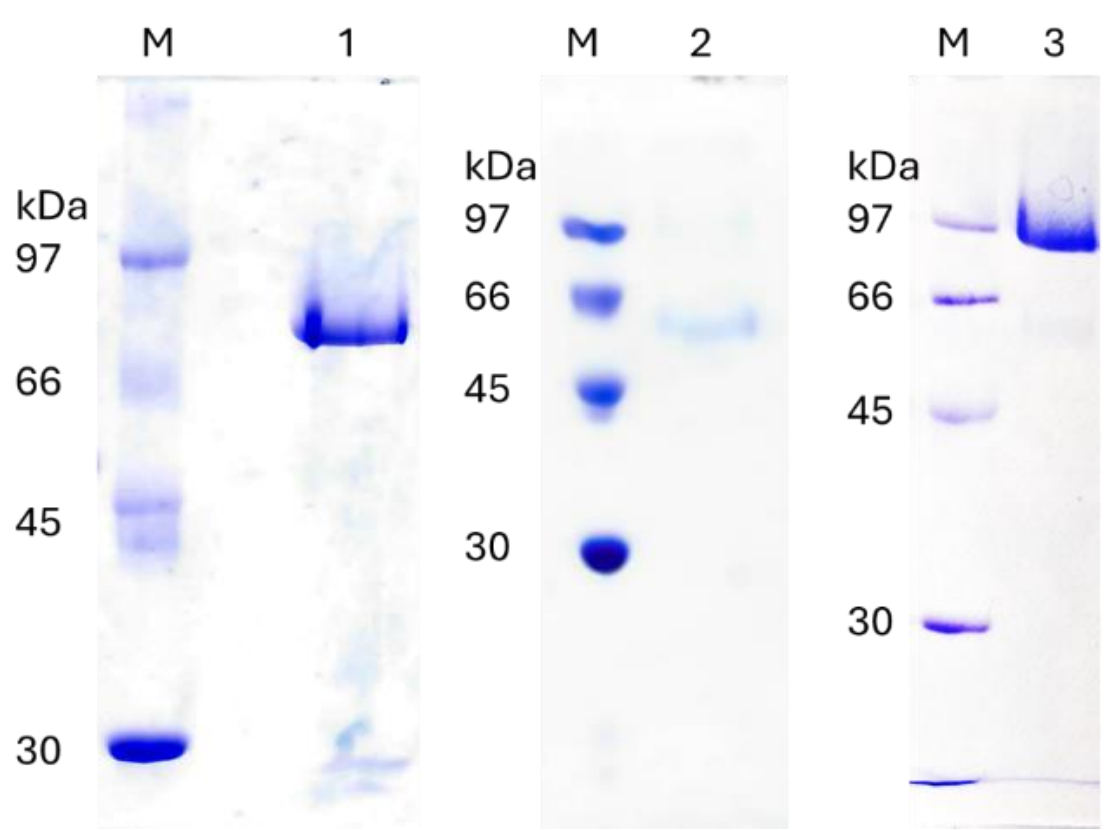

Figure S2. SDS-PAGE analysis of recombinant α-L-fucosidases after purification by ion exchange chromatography. Lane M, molecular mass marker of phosphorylase b from rabbit muscle (97 kDa), bovine serum albumin (66 kDa), chicken egg white ovalbumin (45 kDa), carbonic anhydrase from bovine erythrocytes (30 kDa); lane 1, purified *Fg*FCO1; lane 2, purified *Fo*FCO1; lane 3, purified *Aph*Fuc.

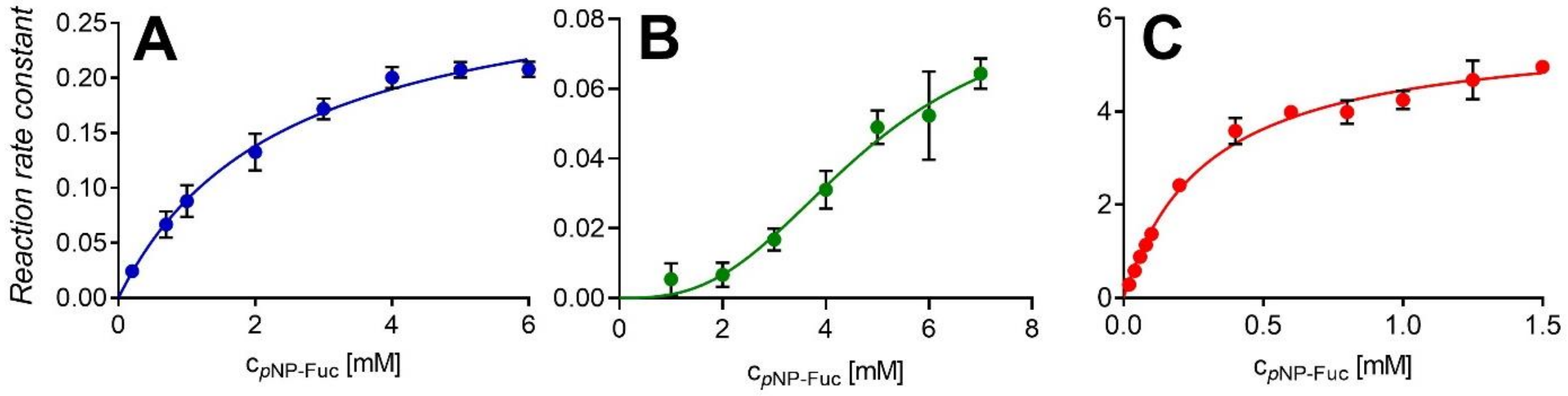

Figure S3. Michaelis-Menten kinetic curves of hydrolysis of *p*NP-α-L-Fuc by recombinant α-L-fucosidases. (**A**), *Fo*FCO1; (**B**), *Fg*FCO1; (**C**), *Aph*Fuc. Kinetic curves were measured by end-point kinetic assay. The kinetic parameters (K_M, k_{cat}, K_{half}) were extracted by non-linear regression using GraphPad Prism 9 (GraphPad Software, USA).

4.3. Analytical Transfucosylation Reactions

Transglycosylation reactions were tested on an analytical scale (100 μL) and screened by TLC (*i*PrOH:H_2O:NH_4OH *aq*., 7:2:1, *v/v/v*), and HPLC (Section 2.1). All reactions contained 30 mM donor *p*NP-α-L-Fuc, and 100-500 mM acceptor. The following acceptors were tested: D-galactose (**5**), arabinose (**6**), L-fucose (**7**), L-rhamnose (**8**); disaccharides: lactose (**9**), rutinose (**10**); and modified saccharides: lactosyl-*t*Boc (**11**), *p*NP-β-D-galactoside (**12**), *p*NP-β-lactoside (**13**), *p*NP-β-melibioside (**14**). The reactions ran in McIlvaine buffer pH 5.0 and were started by adding 0.3 U, 0.3 mU or 3 mU of *Fo*FCO1, *Fg*FCO1 or *Aph*Fuc, respectively. They ran for up to 24 h, until almost all donor was consumed.

5. Molecular Modeling of *Aph*Fuc and Docking Analysis

Molecular modeling of *Aph*Fuc was performed using AlphaFold3 *via* the Foldify platform (https://foldify.cloud.e-infra.cz). To investigate interactions relevant to transglycosylation, the enzyme was modeled in the complex with the product of the autocondensation reaction, *p*-nitrophenyl α-L-fucopyranosyl-(1→2)-α-L-fucopyranoside (**15**). Due to the inherent difficulty of reliably modeling the transglycosylation acceptors in the presence of the α-L-fucosyl-enzyme intermediate, particularly with respect to the dynamic nature of the reaction coordinate, the product molecule was used as a proxy to evaluate potential acceptor binding interactions. The initial placement of the substrate was guided by structural alignment with a homologous α-L-fucosidase containing a bound inhibitor (PDB ID: 2ZX7),[8] followed by refinement using the JAMDA web server (https://proteins.plus).[9] Protein-ligand interactions were analyzed using PoseView[10, 11] and visualized in PyMOL (Schrödinger, LLC), and aromatic residues within 3 Å of the ligand were identified. The docking simulations of *p*NP-carrying acceptors, *p*NP-α-L-Fuc (**1**), *p*NP-β-D-galactoside (**12**), *p*NP-β-lactoside (**13**), and *p*NP-β-melibioside (**14**), were performed using the JAMDA web server.[9] Initial ligand placement was guided by structural alignment with homologous α-L-fucosidase from *Thermotoga maritima* (PDB ID: 2ZX7).[8] The resulting complexes were subjected to energy minimization in YASARA to remove steric clashes.

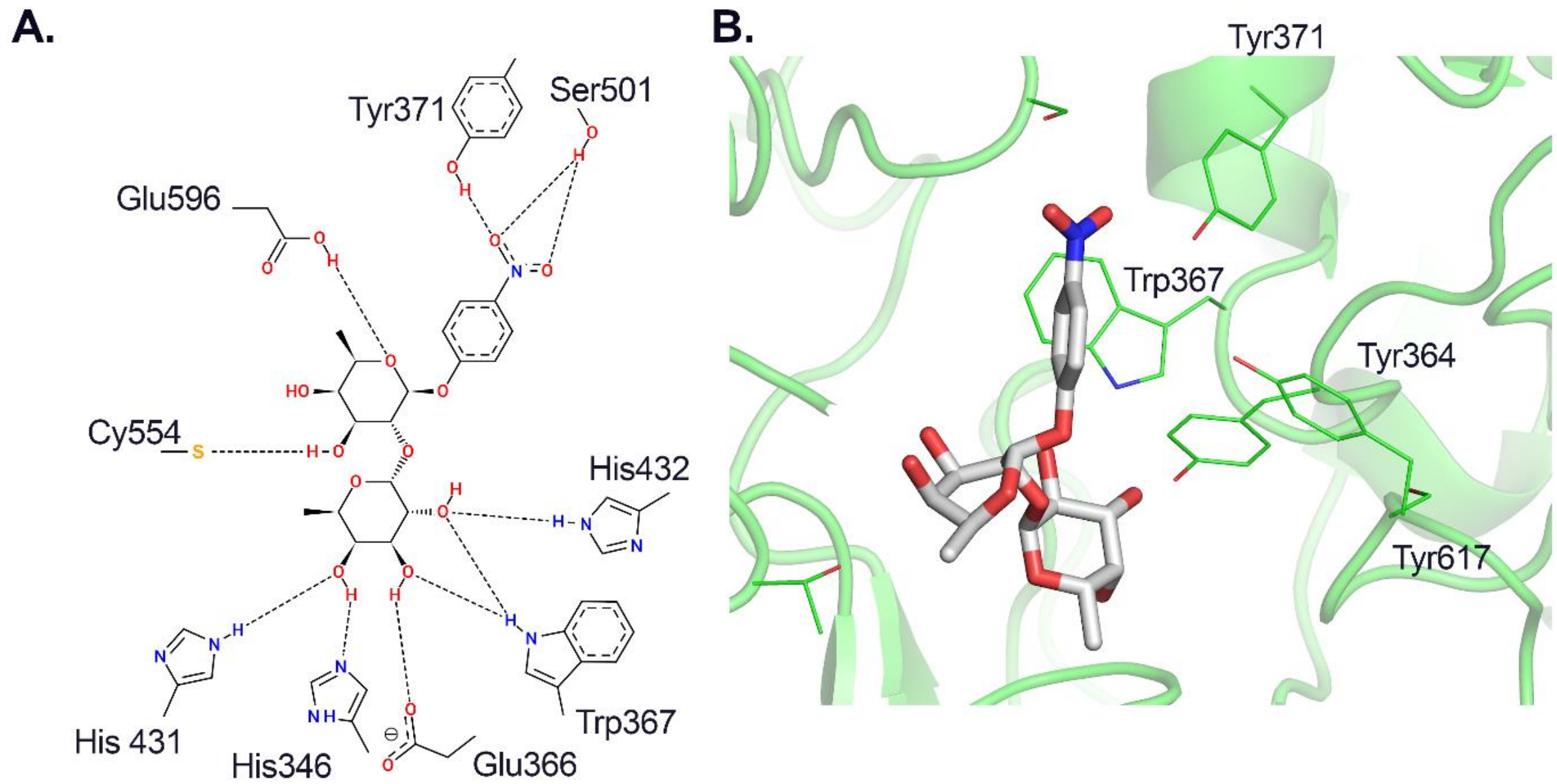

Figure S4. A. Hydrogen bonding interaction of autocondensation product **15** in the active site of *Aph*Fuc. **B.** Aromatic residues within 3 Å from product **15**. The catalytic residue Glu596 is positioned to act as a general acid/base and may contribute to the stabilization of the α-L-fucosyl-enzyme intermediate.

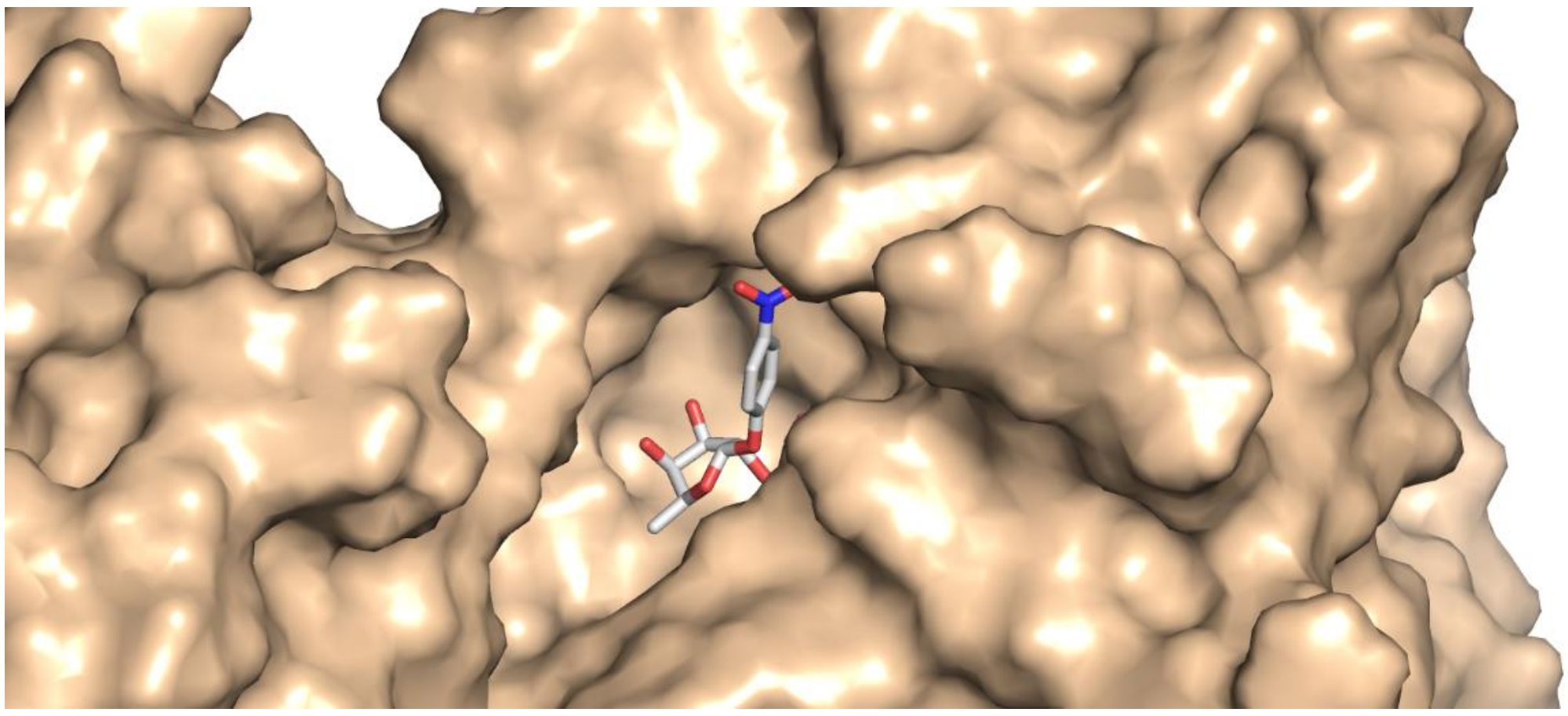

Figure S5. *Aph*Fuc surface with autocondensation product **15** shown as sticks (generated by Pymol).

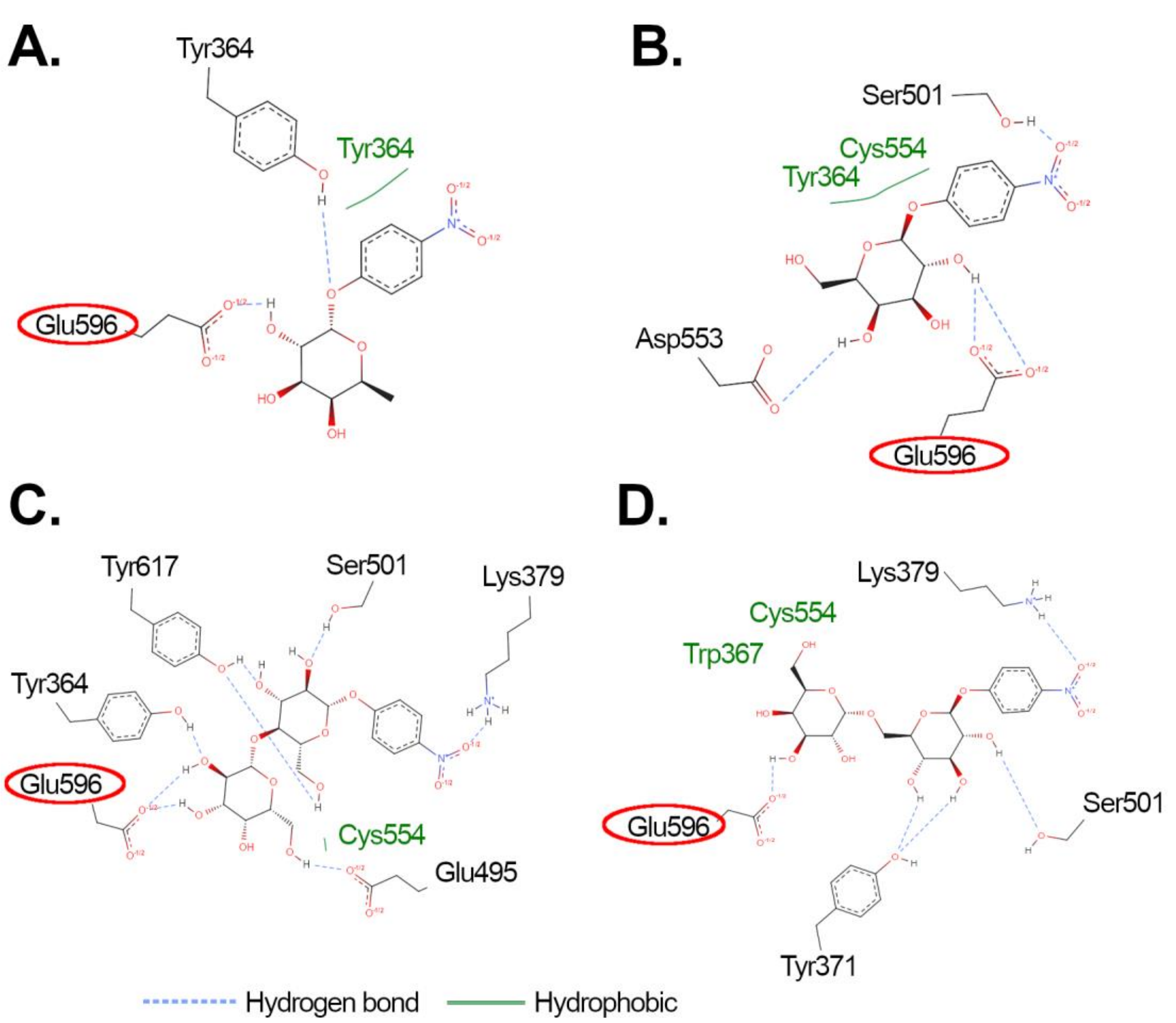

Figure S6. Interactions of selected transglycosylation acceptors docked in the active site of *Aph*Fuc. Catalytic residue Glu596 (highlighted by red circle) acts the catalytic acid/base as interfered from the structural alignment with the homolog from *Thermotoga maritima* (PDB ID: 2ZX7).[8] **A**, *p*NP-α-L-Fuc (**1**); **B**, *p*NP-β-D-galactoside (**12**); **C**, *p*NP-β-lactoside (**13**), and **D**, *p*NP-β-melibioside (**14**), with distances between the nucleophilic hydroxyl group and the anomeric carbon of the glycosyl-enzyme intermediate as follows: ~0.33 nm, ~0.48 nm, ~0.28 nm, and ~0.37 nm for **1**, **12**, **13**, and **14**, respectively.

6. Structural Characterization of Prepared Compounds

6.1. 6-Deoxy-D-glucopyranose

α-anomer: ^{1}H NMR (700 MHz, D_2O) δ 5.23 (d, J = 3.8 Hz, 1H, H-1), 3.95 (dq, J = 9.7, 6.2 Hz, 1H, H-5), 3.71 (t, J = 9.5 Hz, 1H, H-3), 3.60 (dd, J = 9.8, 3.9 Hz, 1H, H-2), 3.20 (t, J = 9.5 Hz, 1H, H-4), 1.31 (d, J = 6.3 Hz, 3H, CH_3) ppm. ^{13}C NMR (176 MHz, D_2O) δ 92.1 (C-1), 75.3 (C-4), 72.6 (C-3), 71.9 (C-2), 67.5 (C-5), 16.9 (C-6) ppm.

β-anomer: ^{1}H NMR (700 MHz, D_2O) δ 4.68 (d, J = 8.0 Hz, 1H), 3.55 (dq, J = 9.5, 6.2 Hz, 1H, H-5), 3.49 (t, J = 9.3 Hz, 1H, H-3), 3.30 (dd, J = 9.3, 7.9 Hz, 1H, H-2), 3.22 (t, J = 9.3 Hz, 3H, H-4), 1.34 (d, J = 6.2 Hz, 3H, CH_3) ppm. ^{13}C NMR (176 MHz, D_2O) δ 95.8 (C-1), 75.6 (C-3), 75.0 (C-4), 74.5 (C-2), 72.0 (C-5), 16.9 (C-6) ppm.

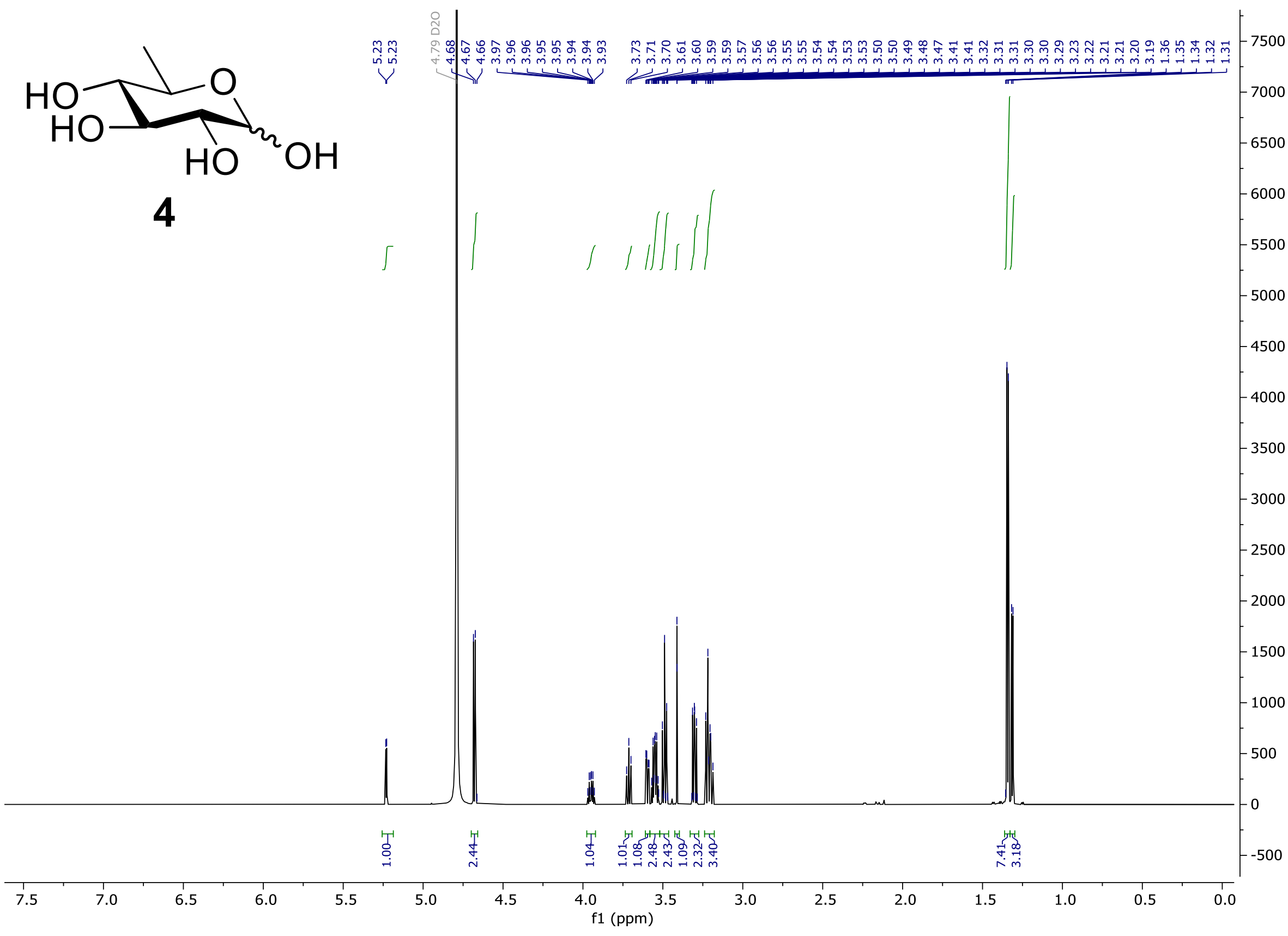

Figure S7. ^{1}H NMR spectrum of compound **4** (700 MHz, D_2O, 30 °C).

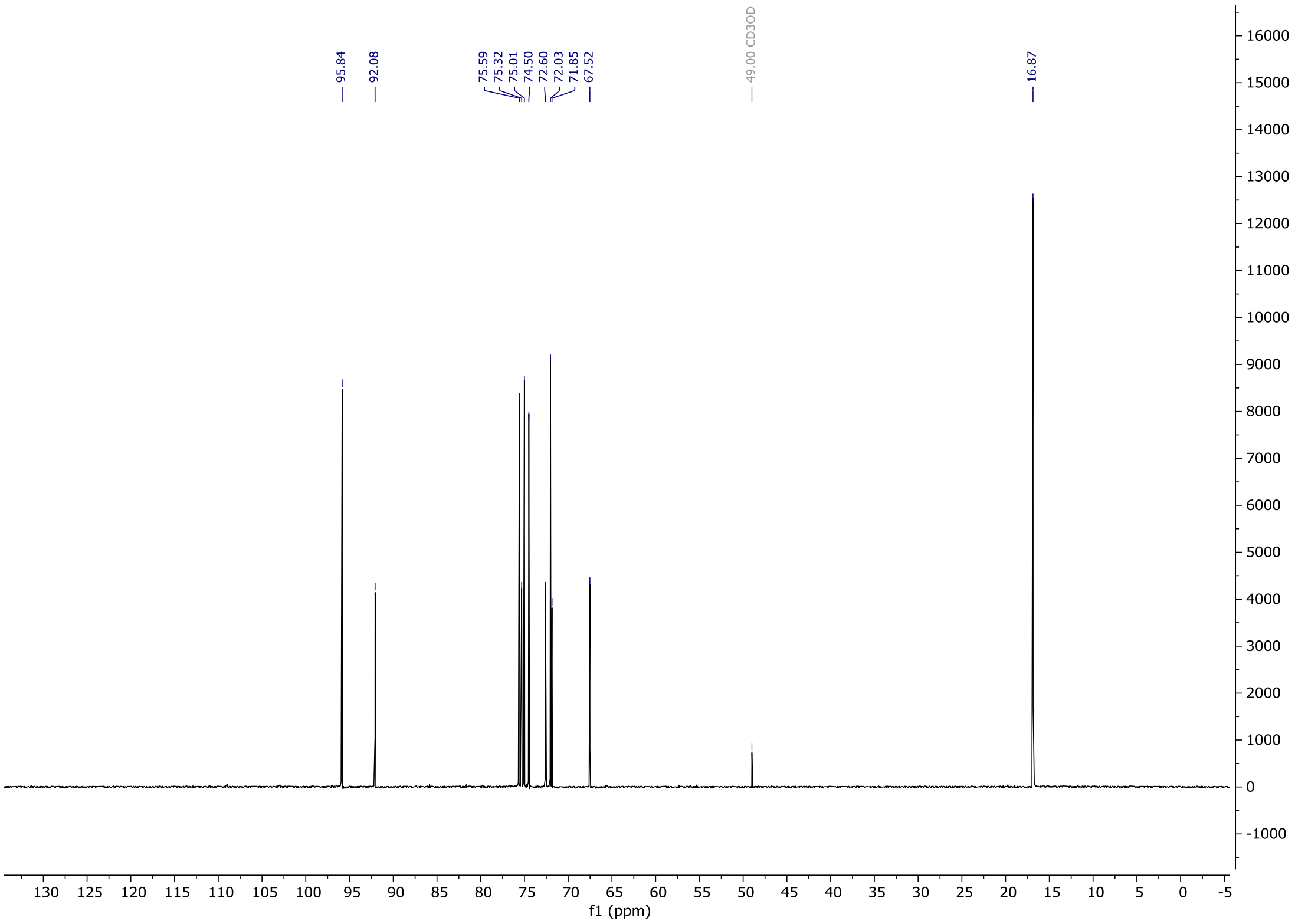

Figure S8. ^{13}C NMR spectrum of compound **4** (176 MHz, D_2O, 30 °C).

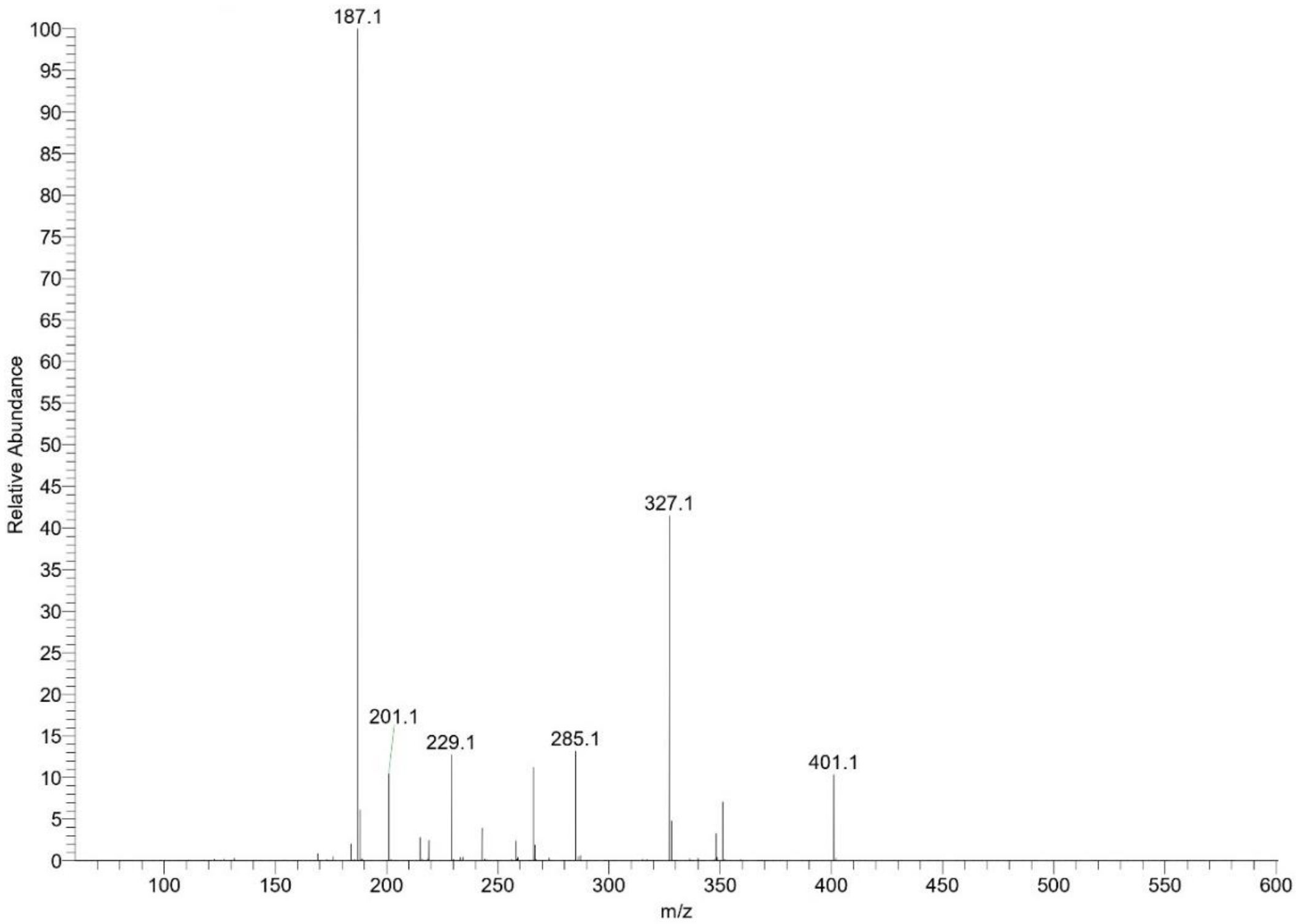

Figure S9. MS spectrum (ESI^+) of compound **4**. $[M+Na]^+$ *m/z* 187.1; $[M+K]^+$ *m/z* 201.1; $[M+Na+CH_3CN]^+$ *m/z* 229.1; $[2M+H]^+$ *m/z* 327.1; $[2M+Na+H_2O+CH_3OH]^+$ *m/z* 401.1. HRMS *m/z*: calcd. for $C_6H_{12}O_5$ $[M+Na]^+$: 187.05769, found 187.05798 (1.54358 ppm).

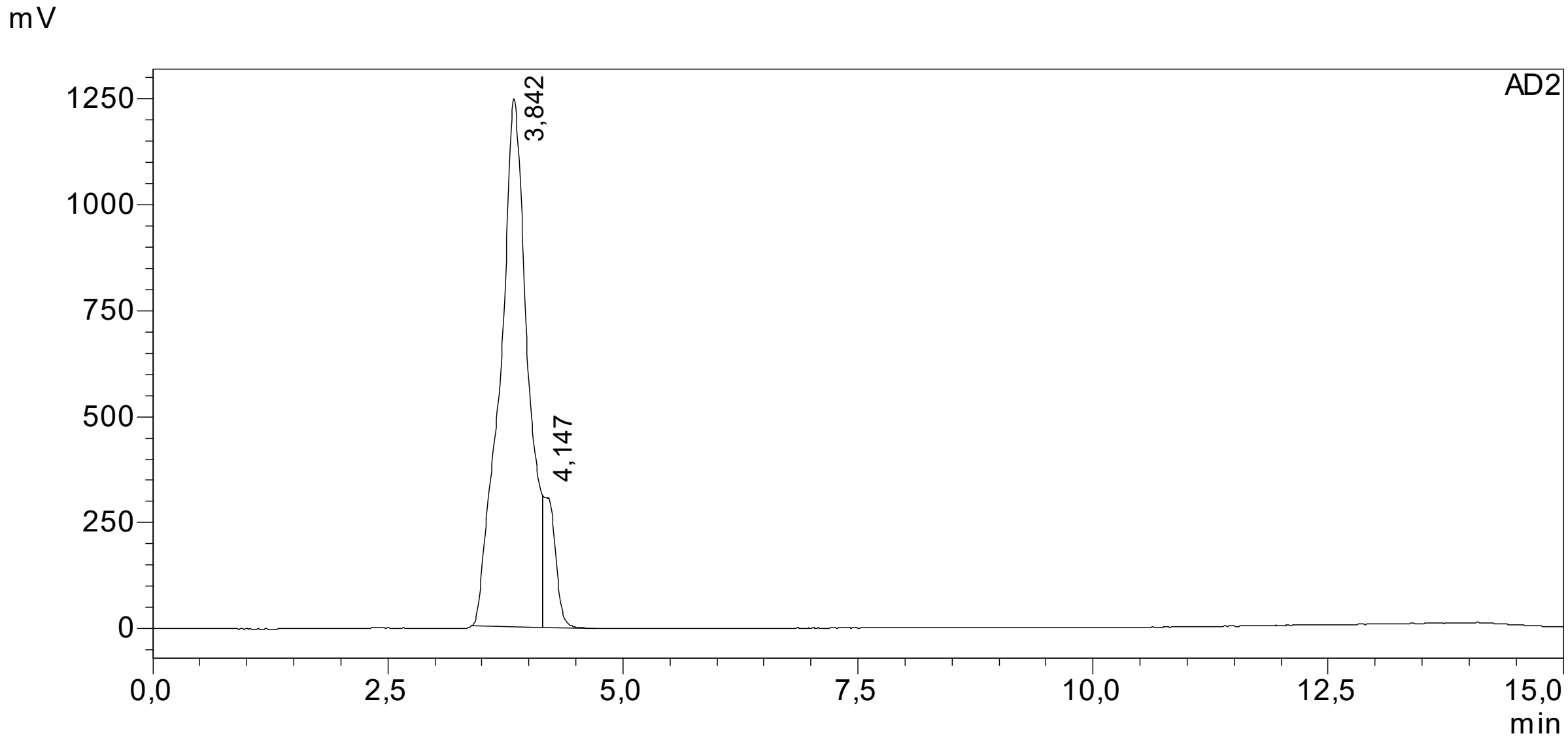

Figure S10. HPLC chromatogram of compound **4** (α/β anomers, 3.842 and 4.147 min; purity 99%).

6.2. *p*-Nitrophenyl α-L-fucopyranosyl-(1→2)-α-L-fucopyranoside

Table S2. ^{1}H and ^{13}C NMR data for compound **15** (600.23 MHz for ^{1}H, 150.93 MHz for ^{13}C, D_2O, 30 °C)

	**Atom**	δ_C	**m.**	δ_H	n_H	**m.**	***J*[Hz]**
**aglycon**	***ipso-***	161.89	s	-	0	-	-
	ortho-	117.23^{x}	d	7.330	2	m	$\Sigma J = 9.3$
	meta-	126.25^{x}	d	8.291	2	m	$\Sigma J = 9.3$
	para-	142.59	s	-	0	-	-
**Fuc^{A}**	**1**	94.53	d	6.024	1	d	3.7
	**2**	72.19	d	4.095	1	dd	10.3, 3.7
	**3**	68.13	d	4.258	1	dd	10.3, 3.4
	**4**	71.89	d	3.955	1	dd	3.4, 0.6
	**5**	68.27	d	4.182	1	dq	0.6, 6.6
	**6**	15.40	q	1.203	3	d	6.6
**Fuc^{B}**	**1**	96.47	d	5.082	1	d	4.0
	**2**	67.96	d	3.777	1	dd	10.4, 4.0
	**3**	69.49	d	3.974	1	dd	10.4, 3.4
	**4**	72.01	d	3.858	1	dd	3.4, 0.9
	**5**	67.28	d	4.334	1	dq	0.9, 6.6
	**6**	15.52	q	1.268	3	d	6.6

^{x} … two carbon atoms

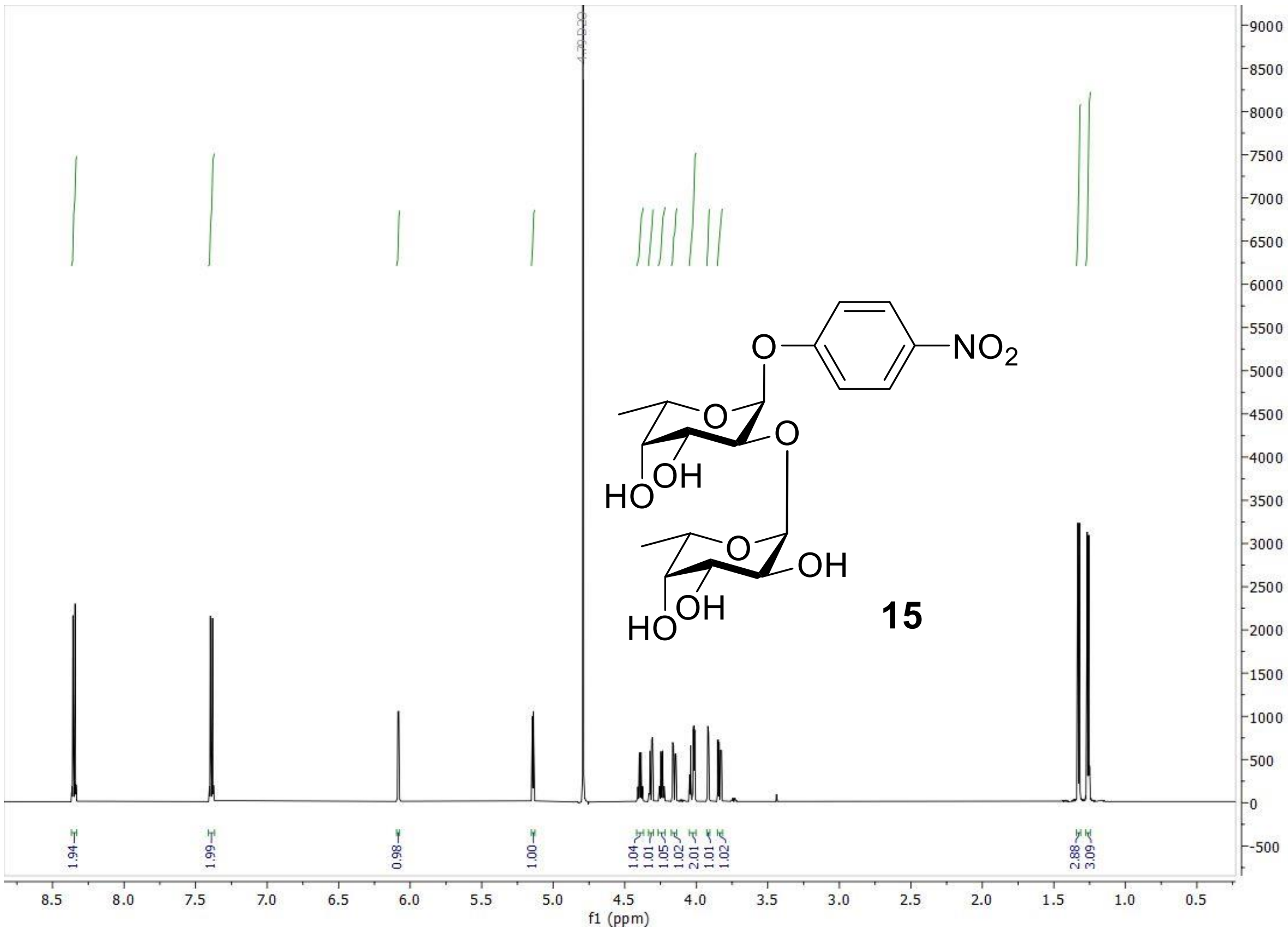

Figure S11. ^{1}H NMR spectrum of compound **15** (600 MHz, D_2O, 30 °C).

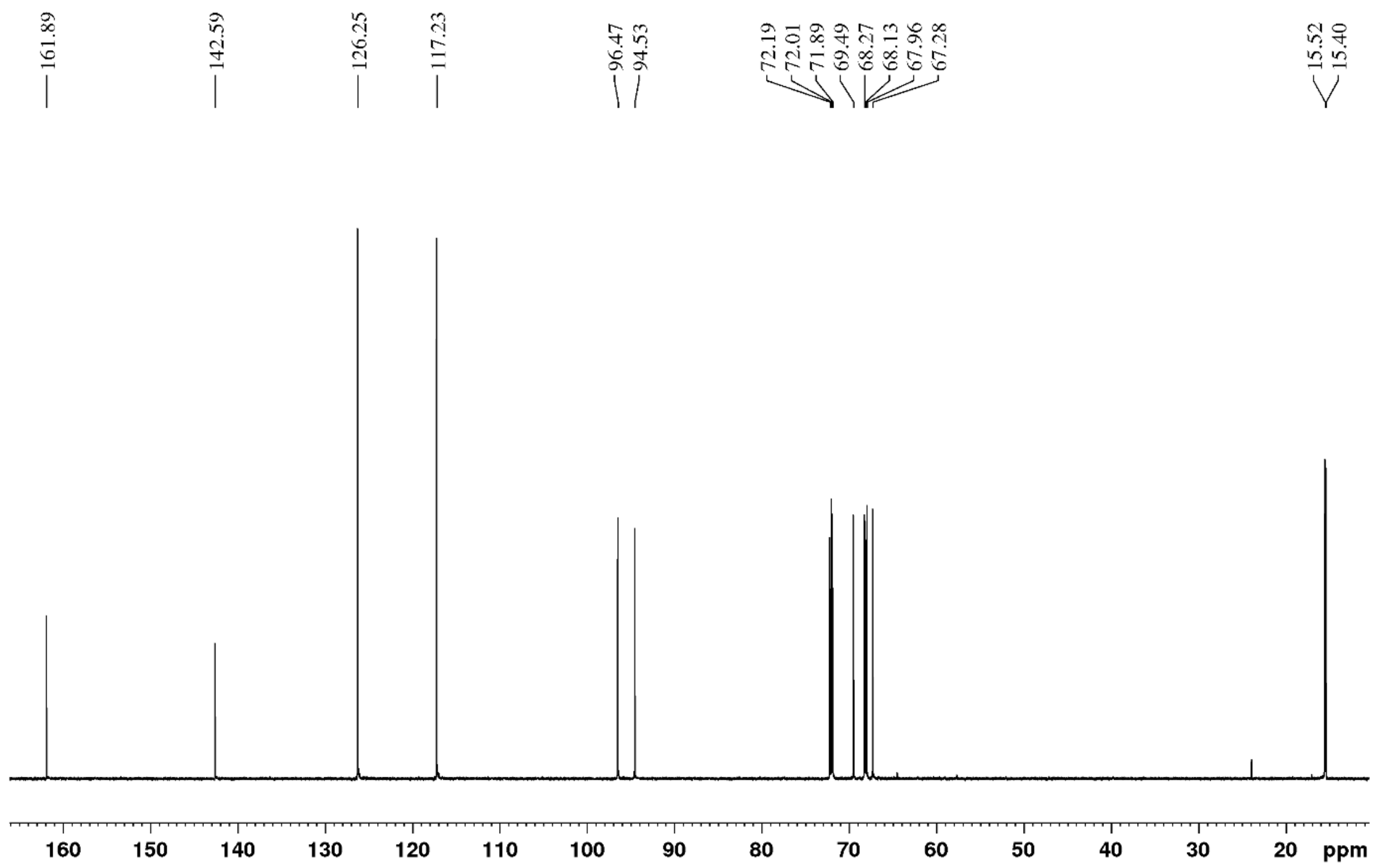

Figure S12. ^{13}C NMR spectrum of compound **15** (150 MHz, D_2O, 30 °C).

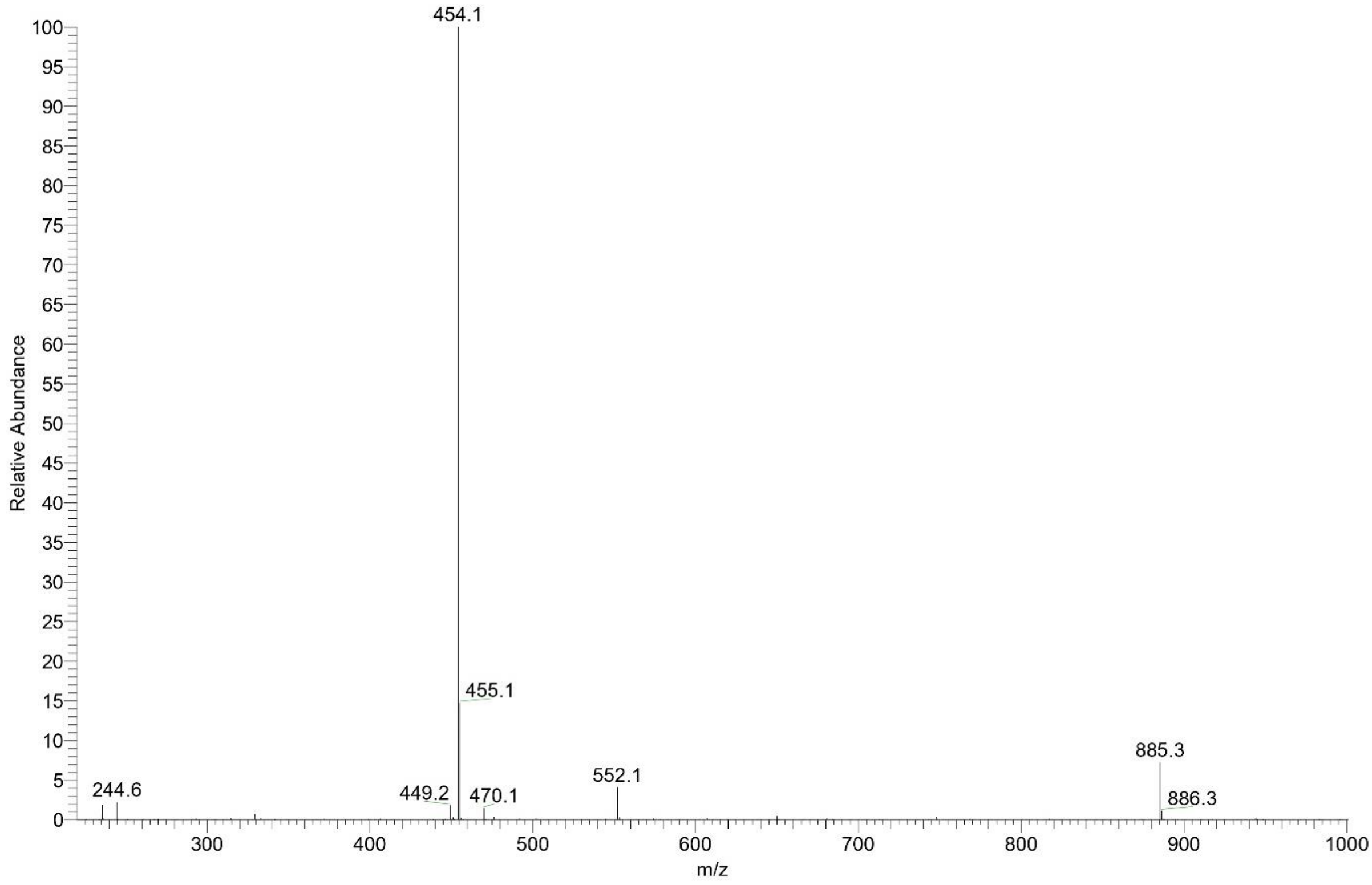

Figure S13. MS spectrum (ESI^+) of compound **15**. $[M+Na]^+$ *m/z* 454.1; $[2M+Na]^+$ *m/z* 885.3. HRMS (ESI^+) *m/z*: calculated for $C_{18}H_{25}O_{11}$ $[M+Na]^+$: 454.13198, found 454.13177 (-0.46076 ppm).

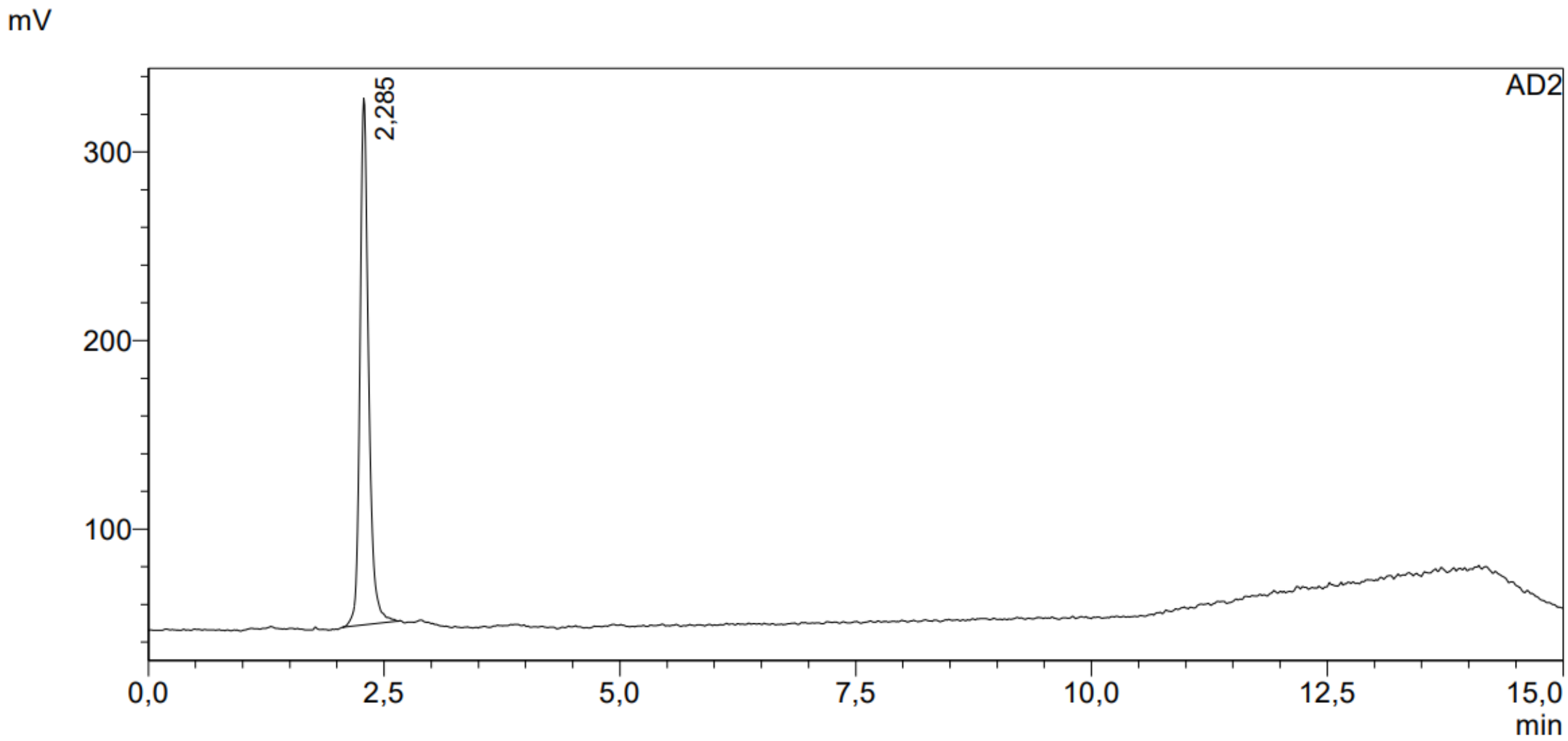

Figure S14. HPLC chromatogram of compound **15** (2.285 min; purity 99%).

7. References

1. Kapešová, J.; Petrásková, L.; Markošová, K.; Rebroš, M.; Kotik, M.; Bojarová, P.; Křen, V., Bioproduction of quercetin and rutinose catalyzed by rutinosidase: Novel concept of "solid state biocatalysis". *Int. J. Mol. Sci.* **2019,** *20* (5), 1112.
2. Šimčíková, D.; Kotik, M.; Weignerová, L.; Halada, P.; Pelantová, H.; Adamcová, K.; Křen, V., α-L-Rhamnosyl-β-D-glucosidase (rutinosidase) from *Aspergillus niger*: characterization and synthetic potential of a novel diglycosidase. *Adv. Synth. Catal.* **2015**, *357* (1), 107–117.
3. Slámová, K.; Červený, J.; Mészáros, Z.; Friede, T.; Vrbata, D.; Křen, V.; Bojarová, P., Oligosaccharide ligands of galectin-4 and its subunits: multivalency scores highly. *Molecules* **2023,** *28* (10), 4039.
4. Ziems, H.; Komabayashi, M.; Lehmann, P.; Villinger, A.; Jopp, S., Structure-activity relationships of glucose-based Pd^{II}-Bis(NHC) complexes in a model Suzuki-Miyaura reaction. *Eur. J. Org. Chem.* **2024,** *27* (21), e202400177.
5. Luo, T.; Guo, Y.-F.; Xu, T.-T.; Dong, H., Visible-light-promoted desulfurization to synthesize deoxyglycosides. *J. Org. Chem* **2023,** *88* (13), 8024–8033.
6. Rönnols, J.; Pendrill, R.; Fontana, C.; Hamark, C.; d'Ortoli, T. A.; Engström, O.; Ståhle, J.; Zaccheus, M. V.; Säwén, E.; Hahn, L. E.; Iqbal, S.; Widmalm, G., Complete ^{1}H and ^{13}C NMR chemical shift assignments of mono- to tetrasaccharides as basis for NMR chemical shift predictions of oligosaccharides using the computer program CASPER. *Carbohydr. Res.* **2013,** *380*, 156–166.
7. Bradford, M. M., A rapid and sensitive method for the quantitation of microgram quantities of protein utilizing the principle of protein-dye binding. *Anal. Biochem.* **1976,** *72* (1-2), 248–254.
8. Wu, H. J.; Ho, C. W.; Ko, T. P.; Popat, S. D.; Lin, C. H.; Wang, A. H., Structural basis of α-fucosidase inhibition by iminocyclitols with K(i) values in the micro- to picomolar range. *Angew. Chem., Int. Ed. Engl.* **2010,** *49* (2), 337-40.
9. Flachsenberg, F.; Ehrt, C.; Gutermuth, T.; Rarey, M., Redocking the PDB. *J. Chem. Inf. Model.* **2024,** *64* (1), 219-237.
10. Stierand, K.; Rarey, M., Drawing the PDB: Protein−ligand complexes in two dimensions. *ACS Med. Chem. Lett.* **2010,** *1* (9), 540-545.
11. Stierand, K.; Rarey, M., From Modeling to medicinal chemistry: Automatic generation of two-dimensional complex diagrams. *ChemMedChem* **2007,** *2* (6), 853-860.
